# Liposomal Honokiol Nanoparticles Attenuate Manganese-Induced Hippocampal Neurotoxicity via NRF2/HO-1 and SIRT1/PGC-1α Pathways: Association with Oxidative Stress, Neuroinflammation, Mitochondrial Dysfunction, and Apoptosis

**DOI:** 10.3390/brainsci16090900

**Published:** 2026-08-22

**Authors:** Raed Al Ruwaili, Ekramy M. Elmorsy, Mohamed M. Abdel-Daim, Eida M. Alshammari, Aly A. M. Shaalan, Ola A. Habotta, Manal S. Fawzy, Mai Salem

**Affiliations:** 1 Department of Internal Medicine, College of Medicine, Northern Border University, Arar 91431, Saudi Arabia; raed.alruwaili@nbu.edu.sa; 2 Center for Health Research, Northern Border University, Arar 73213, Saudi Arabia; ekramy.elmorsy@nbu.edu.sa; 3 Department of Pharmaceutical Sciences, Pharmacy Program, Batterjee Medical College, P.O. Box 6231, Jeddah 21442, Saudi Arabia; abdeldaim.m@vet.suez.edu.eg; 4 Pharmacology Department, Faculty of Veterinary Medicine, Suez Canal University, Ismailia 41522, Egypt; 5 Department of Chemistry, College of Sciences, University of Ha’il, Ha’il 2440, Saudi Arabia; eida.alshammari@uoh.edu.sa; 6 Department of Anatomy, Faculty of Medicine, Jazan University, Jazan 45142, Saudi Arabia; ashaalan@jazanu.edu.sa; 7 Department of Histology and Cell Biology, Faculty of Medicine, Suez Canal University, Ismailia 41522, Egypt; 8 Department of Forensic Medicine and Toxicology, Faculty of Veterinary Medicine, Mansoura University, Mansoura 35516, Egypt; ola_ali@mans.edu.eg; 9 Horus Research Center, Horus University—Egypt (HUE), New Damietta 34518, Egypt; 10 Department of Medical Biochemistry and Molecular Biology, Faculty of Medicine, Mansoura University, Mansoura 35516, Egypt; maisalem@mans.edu.eg

**Keywords:** manganese mining neurotoxicity, honokiol, liposomal nanoformulation, oxidative stress, neuroinflammation, NRF2/SIRT1 signaling

## Abstract

**Highlights:**

**What are the main findings?**

- Manganese exposure is associated with hippocampal oxidative stress, mitochondrial dysfunction, neuroinflammation, neurotransmitter imbalance, and apoptosis, with structural neuronal damage in rats.
- Liposomal honokiol nanoparticles are associated with reversal of Mn-induced alterations, NRF2/HO-1 and SIRT1/PGC-1α signaling, and outperforming free honokiol in neuroprotection.

**What are the implications of the main findings?**

- Targeting NRF2/HO-1 and SIRT1/PGC-1α pathways with liposomal honokiol offers a multi-mechanistic strategy against metal-induced neurotoxicity and related brain disorders.
- Nanoencapsulation of honokiol enhances the therapeutic efficacy, supporting its development as a promising neuroprotective nanomedicine.

**Abstract:**

**Background/Objectives**: Manganese (Mn) is a neurotoxic trace element whose excessive accumulation in the brain can induce hippocampal damage via oxidative stress, mitochondrial dysfunction, neuroinflammation, and apoptosis. This study investigated whether honokiol (HNK) and its liposomal nanoformulation (HNK-LNPs) can ameliorate Mn-induced hippocampal neurotoxicity by modulating key antioxidant and mitochondrial regulatory pathways. **Methods**: Male Wistar rats were subjected to Mn exposure to induce hippocampal neurotoxicity and were treated with HNK or HNK-LNPs. We assessed oxidative status via NRF2/HO-1 signaling, antioxidant defenses (glutathione, GPx, SOD, CAT), and oxidative indices (ROS, MDA). Neuroinflammatory markers (NF-κB, TNF-α, IL-1β, IL-6, Iba-1), mitochondrial respiratory chain function and ATP levels, SIRT1/PGC-1α signaling, and neurotransmitter homeostasis were evaluated. We analyzed apoptosis using Bax, Bcl-2, caspase-3, and cytochrome c, along with histopathological and ultrastructural examination of the hippocampus. **Results**: Mn exposure was associated with NRF2/HO-1 downregulation, depleted endogenous antioxidants, increased ROS and MDA levels, and increased NF-κB–driven neuroinflammation and microglial Iba-1 expression. Mn was further associated with reduced ATP synthesis, dysregulation of SIRT1/PGC-1α signaling, and disrupted neurotransmitter balance, with a pro-apoptotic shift (elevated Bax, caspase-3, cytochrome c; reduced Bcl-2) and neuronal degeneration. Co-treatment with HNK, and more prominently with HNK-LNPs, was associated with reversing these alterations, restoring antioxidant and mitochondrial pathways, dampening inflammatory cascades, normalizing neurotransmitters, and favoring neuronal survival, with many indices approaching control values and consistently surpassing free HNK. **Conclusions**: Liposomal encapsulation significantly enhances honokiol’s neuroprotection against Mn-induced hippocampal neurotoxicity, likely via improved CNS bioavailability and coordinated modulation of NRF2/HO-1 and SIRT1/PGC-1α pathways. These findings support HNK-LNPs as a promising multi-mechanistic therapeutic strategy for metal-induced and related neurotoxic brain disorders.

## 1. Introduction

Manganese (Mn) is an indispensable trace element that participates in a broad spectrum of physiological processes [1]. At homeostatic concentrations, it serves as a cofactor for metalloenzymes, most notably manganese superoxide dismutase (Mn-SOD), arginase, and glutamine synthetase, which collectively regulate oxidative balance, amino acid catabolism, and the biosynthesis of key neurotransmitters [2]. Beyond its enzymatic roles, Mn contributes to neuronal signaling, synaptic transmission, and the structural integrity of brain tissue [3,4]. Its essentiality, however, is counterbalanced by a narrow margin between physiological adequacy and toxicity. Chronic overexposure to Mn, arising from mining operations, occupational inhalation, environmental contamination, or excessive dietary intake, disrupts this balance and precipitates a well-characterized neurotoxic syndrome [5]. Clinically, Mn accumulation in the basal ganglia produces a Parkinsonism-like condition, manifesting as bradykinesia, resting tremor, and locomotor impairments [4,6]. More broadly, excess Mn has been linked to cognitive deterioration, working memory deficits, affective disturbances, and olfactory dysfunction [7]. Epidemiological and experimental evidence further associates elevated Mn burden with susceptibility to neurodegenerative diseases, including Alzheimer’s disease and dementia, in both pediatric and adult populations [5,8]. Workers engaged in manganese mining, ore processing, and smelting activities are a high-risk population, as chronic inhalation of Mn-laden dust is one of the most significant environmental routes of neurotoxic exposure [9,10].

Mn’s neurotoxic mechanisms are multifaceted and deeply interconnected. A central consequence of Mn overload is oxidative stress induced by excessive reactive oxygen species (ROS) generation, coupled with suppression of endogenous antioxidant defenses [11]. This oxidative burden damages neuronal lipids, proteins, and nucleic acids [12,13]. Mitochondria represent a primary cellular target, as Mn accumulates within the mitochondrial matrix and disrupts respiratory chain function, thereby impairing ATP production and amplifying ROS output [5]. Compounding these insults, Mn promotes glutamate excitotoxicity, facilitates protein misfolding and aggregation, and activates pro-inflammatory signaling cascades [5,14]. Dysregulated autophagy and mitophagy, endoplasmic reticulum stress, and apoptotic cell death represent additional downstream consequences that converge to produce irreversible hippocampal neurodegeneration [12,15].

Among the molecular targets disrupted by Mn, the NRF2/HO-1 antioxidant pathway and the SIRT1/PGC-1α mitochondrial biogenesis axis have emerged as pivotal determinants of neuronal vulnerability [16]. NRF2 (nuclear factor erythroid 2-related factor 2) orchestrates the transcriptional upregulation of cytoprotective enzymes, and its suppression by Mn compromises the cell’s capacity to neutralize oxidative insults. Simultaneously, SIRT1-dependent activation of PGC-1α governs mitochondrial biogenesis and quality control; its attenuation under Mn exposure amplifies bioenergetic failure and apoptotic commitment [5,17]. Pharmacological restoration of these pathways, therefore, represents a rational and mechanistically grounded therapeutic strategy [5].

Honokiol (HNK), a bioactive biphenolic compound isolated from the bark and seeds of Magnolia officinalis, has attracted growing attention as a multi-target neuroprotective agent [18]. Its capacity to scavenge free radicals, inhibit NF-κB-driven inflammatory signaling, and modulate cellular stress responses renders it pharmacologically well-suited for counteracting Mn-induced neuronal injury [19,20,21]. Nonetheless, the clinical translation of honokiol is constrained by its inherent physicochemical limitations, notably poor aqueous solubility, limited oral bioavailability, and rapid hepatic metabolism, which collectively limit the achievement of therapeutically relevant concentrations in the central nervous system [21].

Nanotechnology-based drug delivery systems offer a compelling solution to these pharmacokinetic barriers. Among these platforms, nanoliposomes, phospholipid bilayer vesicles capable of encapsulating hydrophobic payloads, have demonstrated particular utility in enhancing compound stability, prolonging systemic circulation, and facilitating blood–brain barrier (BBB) penetration [22]. Encapsulation within a liposomal matrix protects the cargo from premature degradation, modifies its release profile, and improves tissue-specific bioavailability [22,23].

Against this background, the present study was designed to evaluate whether honokiol-loaded nanoliposomes (HNK-LNPs) confer more neuroprotection relative to free HNK against Mn-induced hippocampal neurotoxicity in male Wistar rats, a model selected based on its well-established susceptibility to Mn-induced basal ganglia and hippocampal neurodegeneration and its translational relevance to human manganism observed in occupationally exposed workers. The assessment encompassed oxidative stress parameters, inflammatory mediators, mitochondrial respiratory function, neurotransmitter profiles, and the molecular expression of NRF2/HO-1 and SIRT1/PGC-1α signaling components. Histopathological and ultrastructural analyses corroborated the biochemical findings. The authors hypothesized that liposomal encapsulation of honokiol would enhance its neuroprotective efficacy against Mn-induced hippocampal injury by improving CNS bioavailability and enhancing the NRF2/HO-1 and SIRT1/PGC-1α cytoprotective pathways. This investigation aims to advance mechanistic understanding of Mn neurotoxicity and provide experimental evidence supporting nanoformulated honokiol as a viable neuroprotective strategy for mining workers and other populations at risk of Mn overexposure.

## 2. Materials and Methods

### 2.1. Preparation of Honokiol-Loaded Liposomal Nanoparticles (HNK-LNPs)

Honokiol, cholesterol, and L-α-phosphatidylcholine (L-PC) were obtained from Sigma-Aldrich (St. Louis, MO, USA). We prepared liposomal nanoparticles by the thin-film hydration technique at a final honokiol concentration of 10 mg/mL. Briefly, 70 mg of L-PC (0.09 mmol) and 30 mg of cholesterol (0.078 mmol) were dissolved in 10 mL of chloroform/methanol (2:1, *v*/*v*) in a round-bottom flask to yield a clear, homogeneous lipid solution. The organic solvents were removed under reduced pressure using a rotary evaporator, producing a uniform thin lipid film on the flask wall. The resulting film was hydrated with 5 mL of phosphate-buffered saline (PBS), pH 7.4 (cat no. 221864, FT-IR grade, ≥99%, Sigma-Aldrich) containing 1 mg honokiol (approximately 5 μmol), yielding multilamellar vesicles. The suspension was gently agitated at 37 °C for 30 min to promote uniform drug incorporation predominantly within the lipid bilayer, thereby minimizing honokiol partitioning into the aqueous phase. Multilamellar vesicles were subsequently converted to unilamellar nanosized structures by probe sonication using a Vibra-Cell sonicator (Sonics & Materials, Inc., Newtown, CT, USA) at 30% amplitude, with 10-s pulse intervals for a total duration of 10 min; all steps were carried out on ice to avoid thermal destabilization. We removed unencapsulated honokiol by centrifugation at 15,000× *g* for 30 min, and reserved the recovered supernatant for physicochemical characterization.

Particle size (Z-average), polydispersity index (PDI), and zeta potential of HNK-LNPs were determined using dynamic light scattering in triplicate to evaluate colloidal dimensions, surface charge, and formulation homogeneity. High-resolution transmission electron microscopy (JEOL JEM-2100, Tokyo, Japan) was used to examine ultrastructural morphology at an accelerating voltage of 160 kV.

### 2.2. Encapsulation Efficiency and Drug Loading

The unencapsulated fraction of honokiol in the post-centrifugation supernatant was quantified spectrophotometrically at 260 nm (UV-Vis spectroscopy, model UV-1800, Shimadzu Corporation, Kyoto, Japan). Encapsulation efficiency (EE%) and drug loading (DL%) were calculated according to the following equations:

Drug loading (DL, % *w*/*w*) = [mass of encapsulated HNK (mg)/total lipid mass (mg)] × 100

Encapsulation efficiency (EE, %) = [mass of encapsulated HNK (mg)/mass of HNK initially added (mg)] × 100

### 2.3. In Vitro Drug Release

The release kinetics of honokiol from HNK-LNPs were assessed using the dialysis bag diffusion method and compared with those of free honokiol solution. Equal volumes of each formulation, adjusted to an equivalent drug concentration, were placed in dialysis bags (molecular weight cut-off: 12–14 kDa) and immersed in 50 mL PBS (pH 7.4) maintained at 37 ± 0.5 °C under continuous gentle stirring. At scheduled time points, 2 mL aliquots were withdrawn from the release medium and replenished with an equal volume of fresh PBS to preserve sink conditions. Released honokiol was quantified at 260 nm by UV-Vis spectroscopy, and cumulative release percentages were plotted over time.

### 2.4. Fourier-Transform Infrared (FTIR) Spectroscopic Analysis

FTIR spectroscopy was employed to investigate potential physicochemical interactions between honokiol and the lipid matrix components. Powdered samples of free honokiol, L-PC, cholesterol, and lyophilized HNK-LNPs were individually analyzed over the spectral range of 4000–400 cm^−1^. Peak positions were compared across samples to identify hydrogen-bonding interactions, shifts in functional-group vibrations, or structural modifications arising from encapsulation.

### 2.5. Experimental Animals

Sixty healthy adult male (8–10 weeks) Wistar rats (body weight: 190–230 g) were obtained from the Medical Experimental Research Center (MERC), Faculty of Medicine, Mansoura University, Egypt. Male rats were chosen to eliminate the effect of the cyclical hormonal changes among female rats on the study assay outcomes. Animals were housed in groups of five per standard polypropylene cage (48 × 27 × 20 cm) with wood-chip bedding under controlled laboratory conditions: ambient temperature of 25 ± 2 °C, relative humidity of 50 ± 5%, and a 12 h light/dark cycle. No additional environmental enrichment was provided, as the study did not require behavioral assessment. Upon arrival, rats underwent a 2-week acclimatization period with unrestricted access to standard rodent chow and potable water. The standard diet consisted of “Laboratory Rodent Diet 5001”, containing 23% crude protein, 4.5% crude fat, 56% carbohydrates, 6% crude fiber, 2.5% minerals, and 8% ash, with a total energy value of 3.36 kcal/g (Al Wadi Co., Giza, Egypt).

Health status was monitored throughout. Animals were inspected daily for clinical signs of distress, pain, abnormal behavior, or morbidity. Body weight loss exceeding 20% of initial body weight, or the occurrence of severe neurological signs precluding normal feeding and ambulation, was pre-established as the humane endpoint criterion necessitating immediate euthanasia; this threshold was not reached in any animal during the study period. No unexpected adverse events or mortalities were recorded throughout the experimental protocol. All experimental procedures were conducted in accordance with OECD Guidelines for oral toxicity testing and were approved by the institutional “Animal Care and Use Committee, Faculty of Veterinary Medicine, Mansoura University, Egypt, registration code number MU-ACUC; VM.R.26.05.292.”

### 2.6. Experimental Design

Sixty rats were randomly allocated using a computer-generated random number sequence (Microsoft Excel RAND function) into six groups of ten animals each:

- Group I (Control): received the vehicle (30% ethanol) intraperitoneally (i.p.).
- Group II (HNK): received free honokiol at 20 mg/kg i.p. [24]
- Group III (HNK-LNPs): received honokiol-loaded liposomal nanoparticles at an equivalent dose of 20 mg/kg i.p.
- Group IV (Mn): received manganese chloride (MnCl_2_) at 50 mg/kg i.p., twice weekly for four consecutive weeks, to induce neurotoxicity.
- Group V (Mn + HNK): received MnCl_2_ (50 mg/kg) combined with free HNK (20 mg/kg); HNK was administered 1 h prior to MnCl_2_.
- Group VI (Mn + HNK-LNPs): received MnCl_2_ (50 mg/kg) combined with HNK-LNPs (20 mg/kg equivalent); HNK-LNPs were administered 1 h prior to MnCl_2_.

Sample size of n = 10 per group was based on prior studies using similar Mn neurotoxicity rat models; no formal power calculation was performed [25,26]. No animals were excluded from analysis (e.g., died or were removed) during the course of the study. All injections were administered i.p. twice weekly for four weeks. The study employed a preventive/protective experimental paradigm, with HNK or HNK-LNPs administered 1 h before MnCl_2_ exposure. Accordingly, the present design evaluated protection against Mn-induced neurotoxicity rather than therapeutic efficacy after established Mn exposure, and no post-Mn treatment groups were included. The administered HNK dose was equivalent in the free HNK and HNK-LNP groups (20 mg/kg). For HNK-LNPs, the amount of liposomal formulation administered was calculated according to the experimentally determined drug loading to provide an HNK-equivalent dose of 20 mg/kg, thereby allowing direct comparison between the free and nanoformulated treatments. HNK-LNPs were prepared in 30% ethanol to standardize the vehicle across groups. Body weights were recorded weekly, and MnCl_2_ doses were adjusted accordingly. The study was specifically designed to investigate hippocampal neurotoxicity; therefore, potential effects of HNK or HNK-LNPs on peripheral tissues were not evaluated. Male rats were selected to reduce sex-related biological variability and maintain consistency with the experimental model; consequently, sex-dependent treatment responses were beyond the scope of the present study.

### 2.7. Tissue Sample Processing

Prior to euthanasia, rats were fasted for 10 h. Anesthesia was induced by inhalation of 5% isoflurane in oxygen for 5 min; the permanent absence of cardiac and respiratory reflexes confirmed death. Brains were immediately extracted, and the cerebral hemispheres were separated. For each animal, one hemisphere was randomly allocated to biochemical/molecular analyses and the contralateral hemisphere to histopathological examination, with hemisphere allocation determined using a computer-generated random sequence. Thus, all ten animals per group contributed to both biochemical/molecular and histopathological assessments (n = 10 biological replicates/group). For biochemical and molecular analyses, the hippocampus was dissected on ice from the designated hemisphere and stored at −80 °C until processing. Hippocampal tissues were homogenized in ice-cold PBS containing a protease inhibitor cocktail and centrifuged at 12,000 rpm for 10–15 min at 4 °C. The clarified supernatant was collected and used for downstream analyses. Each biochemical and molecular assay was performed in duplicate as technical replicates, and the mean of the two measurements was used as the individual animal value for statistical analysis (n = 10/group). The contralateral hemisphere from each animal was processed for histopathological examination (n = 10/group). For transmission electron microscopy, five animals per group were randomly selected from the histopathological cohort (n = 5/group) for ultrastructural assessment.

### 2.8. Determination of Manganese Concentration in Hippocampal Tissue

Manganese (Mn) concentration in hippocampal tissue was determined using atomic absorption spectrophotometry (AAS). Briefly, hippocampal samples were collected, dried in a hot-air oven at 120 °C to a constant weight, and then subjected to acid digestion with concentrated nitric acid (HNO_3_) under controlled heating until a clear solution was obtained. After cooling, the digested samples were diluted with deionized water to the required volume. Mn concentration was measured using an atomic absorption spectrophotometer (AA-7000, Shimadzu Corporation, Kyoto, Japan). Each sample was analyzed in triplicate to ensure analytical reliability, and the coefficient of variation was maintained below 10%. The results were expressed as mg/g dry tissue weight.

### 2.9. Antioxidant Enzyme Activities and Oxidative Stress Markers

Hippocampal activities of superoxide dismutase (SOD), catalase (CAT), and glutathione peroxidase (GPx) were measured using commercial colorimetric kits from BioDiagnostic (Giza, Egypt), according to the manufacturers’ protocols (SOD: Cat. No. 2521; CAT: Cat. No. 2517; GPx: Cat. No. 2524). Absorbance was recorded at 560 nm (SOD), 510 nm (CAT), and 340 nm (GPx). Reduced glutathione (GSH) levels were similarly determined in hippocampal tissue homogenates using a commercial colorimetric kit (BioDiagnostic, Giza, Egypt; Cat. No. GR 25 11) according to the manufacturer’s protocol. The resulting values represent reduced GSH measured in the whole hippocampal homogenate and do not distinguish between intracellular and extracellular GSH pools. Lipid peroxidation was assessed by measuring malondialdehyde (MDA) via the thiobarbituric acid reactive substances (TBARS) assay (BioDiagnostic, Cat. No. MD 2529; absorbance at 532 nm). Intracellular reactive oxygen species (ROS) were quantified in freshly isolated hippocampal homogenates using a fluorometric ROS assay kit (Assay Genie; Dublin, Ireland; Cat. No. MAES0112) based on 2′,7′-dichlorodihydrofluorescein diacetate (DCFH-DA) oxidation. After a 30-min incubation at 37 °C in the dark, fluorescence was measured at 488/525 nm and expressed as relative fluorescence units (RFU). Hippocampal NRF2 and HO-1 protein concentrations were determined in triplicate using rat-specific sandwich ELISA kits (MyBioSource, Inc., San Diego, CA, USA; NRF2: Cat. No. MBS3807961; HO-1: Cat. No. MBS2024438), with absorbance read at 450 nm against standard calibration curves.

### 2.10. Pro-Inflammatory Mediators

Hippocampal concentrations of nuclear factor kappa B (NF-κB), tumor necrosis factor-alpha (TNF-α), interleukin-1 beta (IL-1β), and interleukin-6 (IL-6) were quantified using rat-specific ELISA kits: NF-κB (MyBioSource, Cat. No. MBS287521), TNF-α (Assay Genie, Cat. No. AEES00516), IL-1β (Assay Genie, Cat. No. RTES00294), and IL-6 (Assay Genie, Cat. No. RTFI00034). Ionized calcium-binding adapter molecule 1 (Iba-1), a marker of microglial activation, was measured using a rat-specific ELISA kit (MyBioSource, Cat. No. MBS3809193). Protein concentrations were normalized using the bicinchoninic acid (BCA) method. All assays were performed in strict accordance with the manufacturers’ instructions, conducted in triplicate, and results were expressed relative to total tissue protein content.

### 2.11. Apoptotic Protein Quantification

Hippocampal levels of Bcl-2 (Cat. No. MBS704498), Bax (Cat. No. MBS2512405), and caspase-3 (Cat. No. MBS261814) were determined using rat-specific ELISA kits from MyBioSource, following the manufacturer’s protocols. Mitochondrial membrane integrity was assessed by measuring cytochrome c release using an ELISA kit from Elabscience Biotechnology Inc. (Houston, TX, USA); results were expressed as nmol/mg protein. All measurements were performed in triplicate.

### 2.12. Mitochondrial Respiratory Chain Complex Activities

Complex I (NADH dehydrogenase) activity was measured as described by Galante and Hatefi [27]. Hippocampal mitochondrial protein (40 μg/mL) was incubated in 40 mM phosphate buffer (pH 7.4) containing 0.1% sodium cholate, 1.5 mM KCN, and 1.3 mM potassium ferricyanide at 30 °C for 1 min. NADH oxidation was initiated by adding 0.14 mM NADH and monitored spectrophotometrically at 340 nm for 1–3 min. Rotenone-sensitive activity was determined by including 1 μM rotenone. Results were expressed as μmol NADH oxidized per minute per mg protein.

Complex II (succinate dehydrogenase) activity was assayed according to King [28]. The reaction mixture (1 mL) contained 0.1 M phosphate buffer (pH 7.4), 0.3 mM EDTA, 20 mM sodium succinate, 0.053 mM 2,6-dichlorophenolindophenol (DCIP), 1 mM KCN, and 20 μL enzyme. Activity was determined from the decrease in absorbance at 600 nm, recorded every 30 s for 2 min.

Complex III (ubiquinol-cytochrome c reductase) activity was determined as described by Green and Ziegler at 38 °C [29]. The 1 mL assay mixture comprised 50 mM phosphate buffer (pH 7.4), 0.1 mM EDTA, 1 mg/mL cytochrome c, 1.6 mM KCN, 1 mM NADH, 10% sodium cholate, and 10–20 μL enzyme. Cytochrome c reduction was followed at 550 nm every 15 s over 2 min, and activity was calculated using an extinction coefficient of 18.5 × 10^3^ cm^2^/mol, expressed as μmol reduced cytochrome c per min per mg protein.

Complex IV (cytochrome c oxidase) activity was measured colorimetrically at 550 nm according to the method of Green and Ziegler [29]. The assay mixture (1 mL) consisted of 50 mM phosphate buffer (pH 7.4), 0.1 mM EDTA, 1 mg/mL cytochrome c, 10% sodium cholate, and 20 μL enzyme. Absorbance was recorded every 15 s for 2 min, and activity was expressed as μmol oxidized cytochrome c per min per mg protein (extinction coefficient: 19.6 mM^−1^·cm^−1^).

### 2.13. Cellular Energy Metabolism

Hippocampal ATP levels were quantified using a commercially available ATP assay kit (Assay Genie, Cat. No. RTEB0950) in accordance with the manufacturer’s instructions. Pyruvate dehydrogenase (PDH) activity was assessed using a PDH activity kit (Assay Genie, Cat. No. RTDL00814). The assay monitors the PDH-catalyzed decarboxylation of pyruvate by measuring the reduction of 2,6-dichlorophenolindophenol (2,6-DCPIP) at 605 nm. One unit of PDH activity represents the reduction of 1 nmol 2,6-DCPIP per minute under the described conditions.

### 2.14. Biogenic Amine Quantification by HPLC-ECD

Hippocampal concentrations of dopamine, norepinephrine, and serotonin were determined by reversed-phase high-performance liquid chromatography coupled with electrochemical detection (HPLC-ECD), following the method of Kim et al. with minor modifications [30]. Twenty microliters of clarified tissue homogenate supernatant were injected onto a Waters HPLC system (Waters Corporation, Milford, MA, USA) equipped with a C18 reverse-phase column (250 × 4 mm, 5 μm). Chromatographic separation was achieved using a mobile phase (pH 4.2) comprising 0.15 M sodium dihydrogen phosphate, 1.75 mM sodium octyl sulfate, 0.25 mM EDTA, and 4% methanol at a flow rate of 1.5 mL/min. Electrochemical detection was performed at +0.800 V, and peak integration was performed using Empower2 software (Waters Corporation, Milford, MA, USA).

### 2.15. Cholinergic Neurotransmission

Hippocampal acetylcholine (ACh) concentrations and acetylcholinesterase (AChE) activity were measured in triplicate using rat-specific ELISA kits from MyBioSource (ACh: Cat. No. MBS8820077; AChE: Cat. No. MBS451136) following the manufacturer’s protocols. Results were expressed relative to total tissue protein content.

### 2.16. SIRT1 and PGC-1α Protein Quantification

Hippocampal concentrations of sirtuin-1 (SIRT1) and peroxisome proliferator-activated receptor γ coactivator-1α (PGC-1α) were determined using rat-specific double-antibody sandwich ELISA kits (MyBioSource; SIRT1: Cat. No. MBS2600246; PGC-1α: Cat. No. MBS2612283). The assay principle involves sequential binding of the target antigen to pre-coated capture antibodies, followed by a biotinylated detection antibody and HRP-avidin conjugate, with TMB substrate yielding a colorimetric signal proportional to protein concentration. Absorbance was recorded at 450 nm, and values were normalized to total protein. All measurements were performed in triplicate.

### 2.17. Gene Expression Analysis by RT-qPCR

Hippocampal mRNA expression of *Nrf2*, *HO-1*, *Sirt1*, and *PGC-1α* was quantified by reverse transcription quantitative PCR (RT-qPCR). Total RNA was isolated using TRIzol reagent (Invitrogen, Thermo Fisher Scientific, Waltham, MA, USA) according to the manufacturer’s instructions. RNA concentration and purity were determined spectrophotometrically to ensure A260/A280 ratios of 1.8–2.0 for downstream analysis. RNA integrity was further confirmed by agarose gel electrophoresis, which demonstrated intact 28S and 18S ribosomal RNA bands with a 28S/18S ratio ≥1.8, indicating adequate RNA quality for gene expression profiling. Complementary DNA synthesis was performed at 42 °C for 60 min using oligo(dT) primers and SuperScript Reverse Transcriptase (Invitrogen, Thermo Fisher Scientific), starting with 1 μg of total RNA in a 12-μL reaction volume, according to the kit protocol (CWBIO, Beijing, China). Genomic DNA contamination was excluded by incorporating no-reverse transcriptase (no-RT) controls in each run; the absence of amplification in all no-RT controls confirmed that the cDNA amplification signals originated exclusively from RNA templates. Quantitative PCR was performed using a TaqMan^®^ Real-Time PCR system (Thermo Fisher Scientific, Waltham, MA, USA) under the following thermal cycling conditions: initial denaturation at 95 °C for 5 min, followed by 45 cycles of 94 °C for 15 s, 60 °C for 30 s, and 72 °C for 30 s, with a final extension at 72 °C for 5 min. The primer sequences used are presented in Table S1. All procedures were in accordance with the updated “minimum information for publication of quantitative real-time PCR experiments (MIQE 2.0)” guidelines [31]. Relative expression was calculated using the 2^−ΔΔCq^ method with Glyceraldehyde-3-phosphate dehydrogenase (*GAPDH*) as the reference gene [32].

### 2.18. Histopathological Examination

Hippocampal tissues were fixed in 10% neutral buffered formalin at a fixative-to-tissue ratio of 20:1 for 72 h at room temperature. Fixed tissues were dehydrated through an ascending ethanol series (70–100%), cleared in xylene, and infiltrated with paraffin at 60 °C for 1 h. Paraffin-embedded specimens were sectioned at 4–5 μm using a rotary microtome, deparaffinized, rehydrated, and stained with hematoxylin and eosin (H&E). Stained sections were dehydrated, cleared in xylene, and cover slipped with a suitable mounting medium for examination under a light microscope.

### 2.19. Ultrastructural Analysis by Transmission Electron Microscopy

Hippocampal fragments were fixed in 2.5% glutaraldehyde in 0.1 M phosphate buffer at 4 °C for 24 h, rinsed thoroughly, and post-fixed in 1% osmium tetroxide for 2 h. Specimens were dehydrated stepwise through graded ethanol (50–100%) followed by acetone, then embedded in epoxy resin. Ultrathin sections (60–70 nm) were cut using an ultramicrotome, double-stained with uranyl acetate and lead citrate, and examined with a JEOL JEM-2100 transmission electron microscope (Akishima, Tokyo, Japan) at 160 kV.

### 2.20. Immunohistochemical Assay of NF-κB

Paraffin-embedded hippocampal sections were deparaffinized in xylene, rehydrated through graded ethanol, and subjected to heat-mediated antigen retrieval in 0.01 M citrate buffer (pH 6.0) for 10 min. Endogenous peroxidase activity was blocked with 3% hydrogen peroxide, followed by blocking with 5% bovine serum albumin for 30 min. Sections were incubated overnight at 4 °C with rabbit monoclonal anti-NF-κB p65 antibody (1:200; Abcam, Cambridge, UK, ab32536), followed by biotinylated goat anti-rabbit secondary antibody (1:500; Abcam) and streptavidin–HRP. Immunoreactivity was visualized using 3,3′-diaminobenzidine (DAB), followed by Mayer’s hematoxylin counterstaining. Negative controls were processed with omission of the primary antibody. Sections were examined at ×400 magnification, and five randomly selected fields per section were analyzed. NF-κB-positive area (%) was quantified using ImageJ v. 1.54 (National Institutes of Health, Bethesda, MD, USA) in a blinded manner.

### 2.21. Statistical Analysis

Investigators responsible for biochemical assays, histopathological scoring, and data analysis were blinded to group allocation throughout the experimental and analytical phases. Group codes were revealed only after all data had been collected and recorded. All data were organized and screened using Microsoft Excel. Normality was verified using the Shapiro–Wilk test, and homogeneity of variance was assessed using the Levene test. Group differences were examined by one-way analysis of variance (ANOVA; SAS version 9.3, SAS Institute Inc., Cary, NC, USA), with Tukey’s post hoc test applied when significant main effects were detected. Results are expressed as mean ± standard error of the mean (SEM). A *p*-value of <0.05 was adopted as the threshold for statistical significance. Graphical representations were produced using GraphPad Prism v9.0 (GraphPad Software, San Diego, CA, USA).

## 3. Results

### 3.1. Physicochemical Characterization of HNK-LNPs

**Figure 1:**
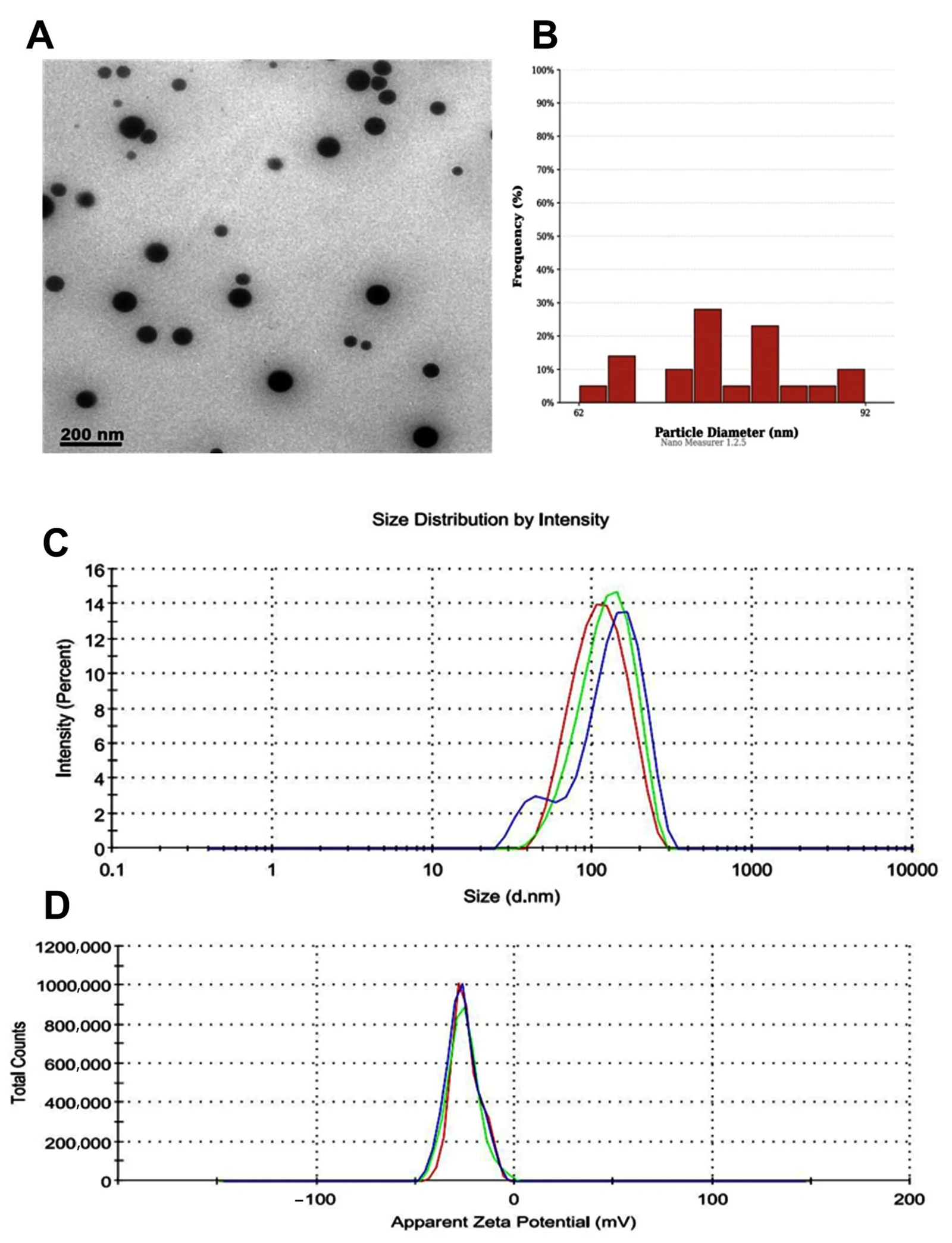
Characterization of honokiol-loaded liposomal nanoparticles; (**A**) TEM micrograph showing nearly spherical particles. (**B**) TEM image-based particle-size distribution histogram, showing particle diameters mainly within the 62–92 nm range. (**C**) DLS size-distribution-by-volume profile. The blue, red, and green curves represent three independent replicate measurements of the same HNK-LNP preparation. (**D**) Zeta potential distribution. The blue, red, and green curves represent three independent replicate measurements of the same HNK-LNP preparation. The Z-average size, polydispersity index (PDI), and zeta potential were 140 nm, 0.262, and −36 mV, respectively.

TEM imaging showed that HNK-LNPs exhibited a predominantly spherical morphology with no prominent aggregation in the examined fields (Figure 1A). Image-based particle-size analysis of the TEM micrographs showed particle diameters predominantly within the 62–92 nm range (Figure 1B). Dynamic light scattering analysis showed a Z-average hydrodynamic diameter of 140 nm, as determined by cumulants analysis, with a PDI of 0.262, indicating a moderately narrow particle-size distribution. The DLS size-distribution-by-volume profile is presented separately in Figure 1C and should not be directly equated with the Z-average diameter because these parameters are derived using different analytical approaches. The zeta potential was −35.41 mV (Figure 1D), indicating a negatively charged particle surface and suggesting electrostatic stabilization under the measurement conditions.

### 3.2. Encapsulation Efficiency and Drug Loading

HNK-LNPs demonstrated efficient drug incorporation within the lipid matrix, with an encapsulation efficiency of 85.65 ± 2.32% and a drug loading capacity of 10.86 ± 1.01% (*w*/*w*), expressed as the mass of encapsulated HNK relative to the total lipid mass. The high encapsulation efficiency is attributable to honokiol’s pronounced hydrophobicity and strong affinity for the phospholipid bilayer, which facilitates preferential partitioning into the lipid phase. These values collectively support the effective incorporation of HNK into the liposomal formulation using the thin-film hydration method.

### 3.3. FTIR Spectroscopic Characterization

**Figure 2:**
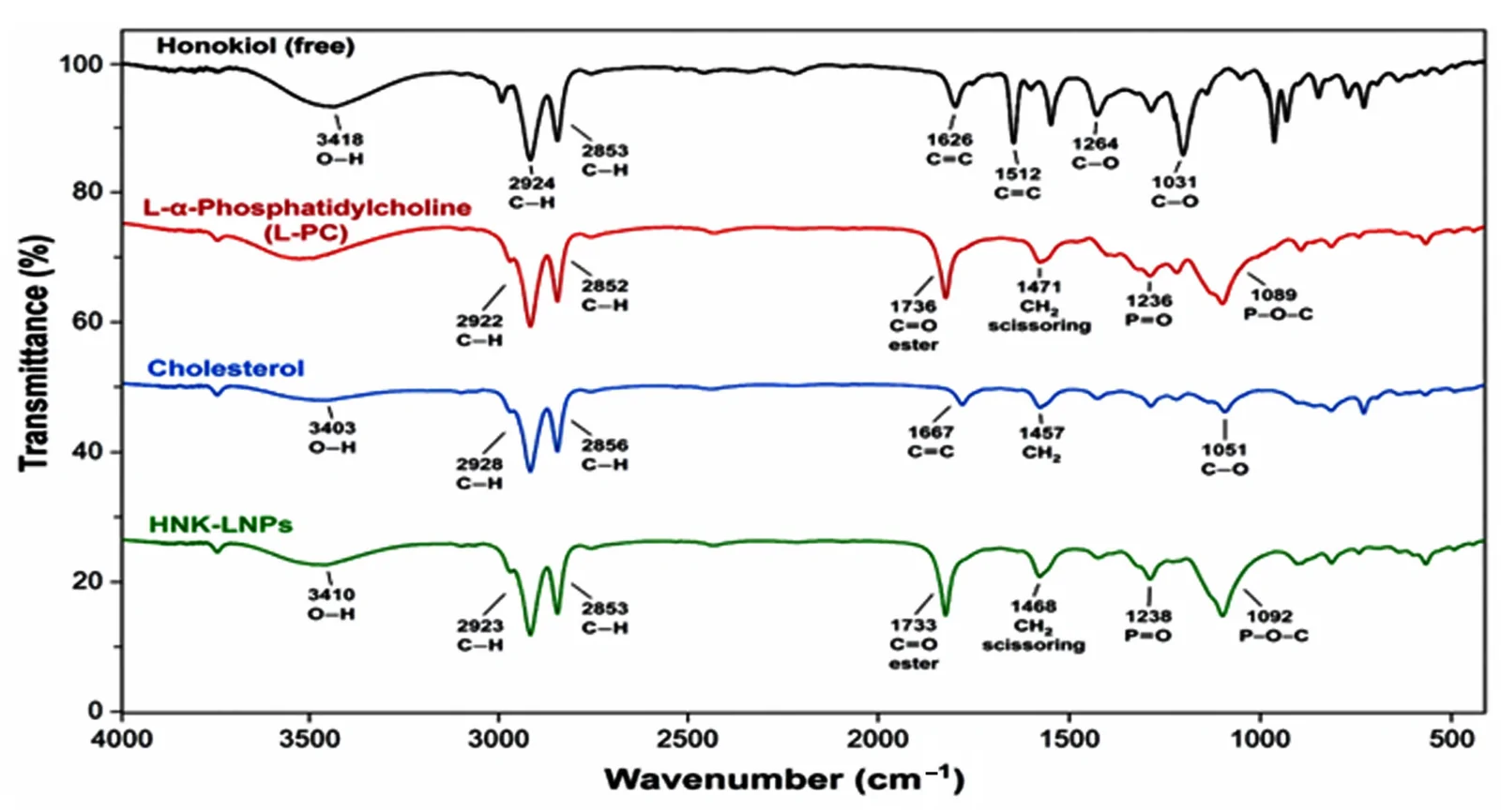
FTIR spectra of free honokiol, L-α-phosphatidylcholine (L-PC), cholesterol, and honokiol-loaded liposomal nanoparticles (HNK-LNPs). The spectra demonstrate the characteristic functional groups of each component, including O–H, C–H, C=O, and P–O–C vibrations. The minor spectral shifts observed in the HNK-LNPs spectrum relative to the individual components confirm successful encapsulation of honokiol within the lipid bilayer and indicate the presence of intermolecular interactions without chemical structural alteration.

FTIR spectra of free honokiol, L-α-phosphatidylcholine (L-PC), cholesterol, and HNK-LNPs are presented in Figure 2. Free honokiol displayed a broad O–H stretching band at approximately 3418 cm^−1^, C–H stretching vibrations at 2924 and 2853 cm^−1^, and aromatic C=C absorptions at 1626 and 1512 cm^−1^. L-PC exhibited a characteristic ester carbonyl (C=O) band at 1736 cm^−1^, together with P=O and P–O–C stretching vibrations at 1236 and 1089 cm^−1^, respectively. Cholesterol presented an O–H stretching band near 3403 cm^−1^ and C–H stretching at 2928 and 2856 cm^−1^. In the HNK-LNPs spectrum, all characteristic component bands were preserved with minor positional shifts; the O–H band appeared at 3410 cm^−1^ and the ester C=O peak at 1733 cm^−1^, indicating successful honokiol incorporation into the lipid bilayer. The retention of principal phospholipid bands with slight displacements, rather than new covalent bond formation, confirms that encapsulation was governed by physical intermolecular interactions, primarily hydrogen bonding and hydrophobic forces, without inducing any chemical structural modification.

### 3.4. In Vitro Release Profile

**Figure 3:**
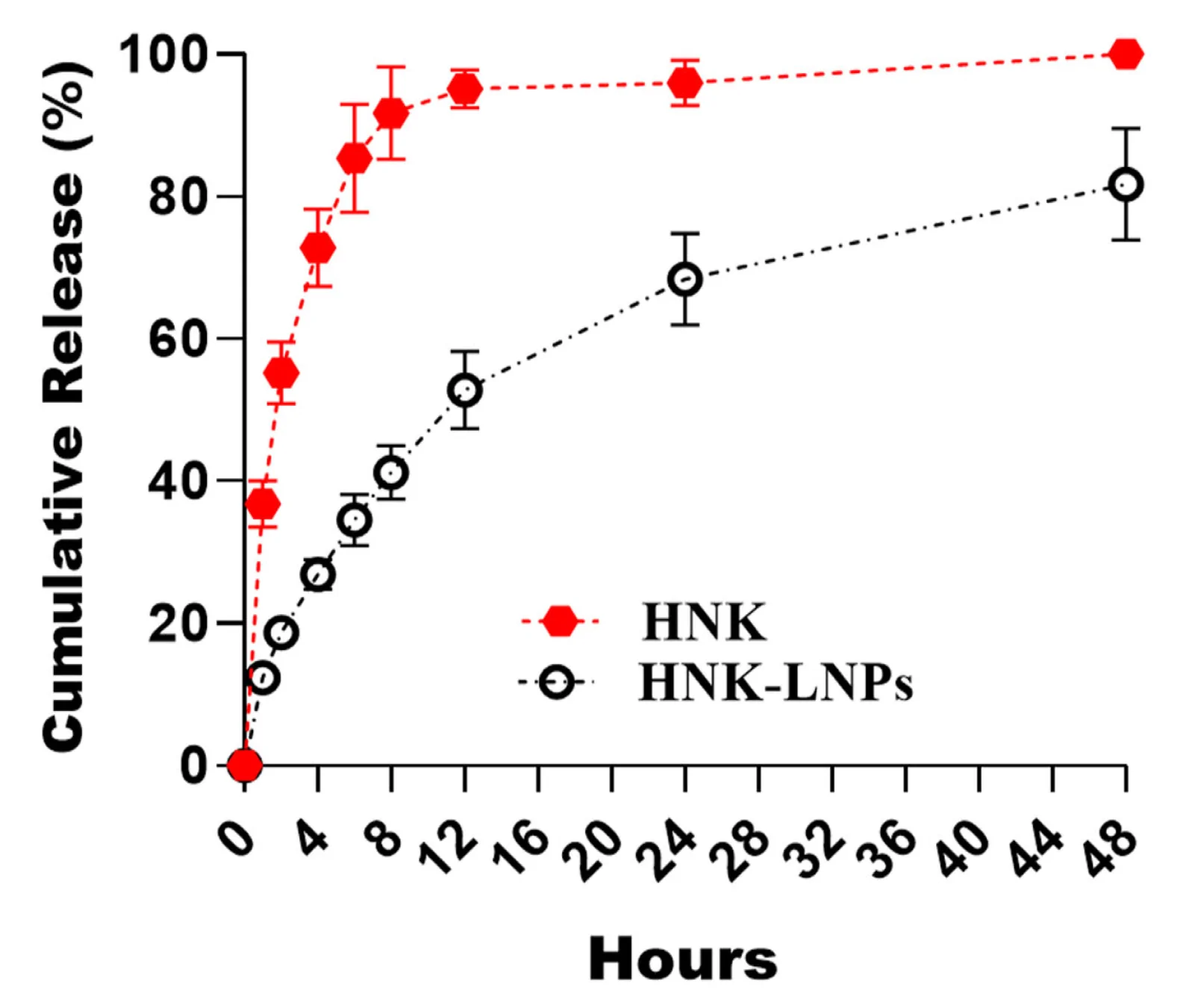
In vitro cumulative release profile of free Honokiol (HNK) and HNK-LNPs in phosphate-buffered saline (PBS, pH 7.4) at 37 °C over 48 h, showing a rapid burst release from free Honokiol compared to a sustained and controlled release pattern from the liposomal formulation. The experiment was independently performed in triplicate (n = 3 independent replicates), with each sample analyzed in three technical replicate wells. Data are presented as mean ± SEM.

The release behavior of free honokiol and HNK-LNPs differed markedly (Figure 3). Free honokiol exhibited a rapid burst release, with over 85% of the drug diffusing across the dialysis membrane within 6 h and near-complete release by 24 h, reflecting uncontrolled diffusion kinetics. By contrast, HNK-LNPs displayed a substantially slower, more sustained release pattern, releasing approximately 34% of the encapsulated drug within the first 6 h, with gradual, progressive release continuing over 72 h, ultimately reaching approximately 90% cumulative release. These findings confirm that liposomal encapsulation markedly prolongs honokiol release relative to the free compound, supporting its utility as a controlled-delivery platform for sustained CNS exposure.

### 3.5. Effect of HNK Formulations on Mn Concentration in Hippocampal Tissue

**Figure 4:**
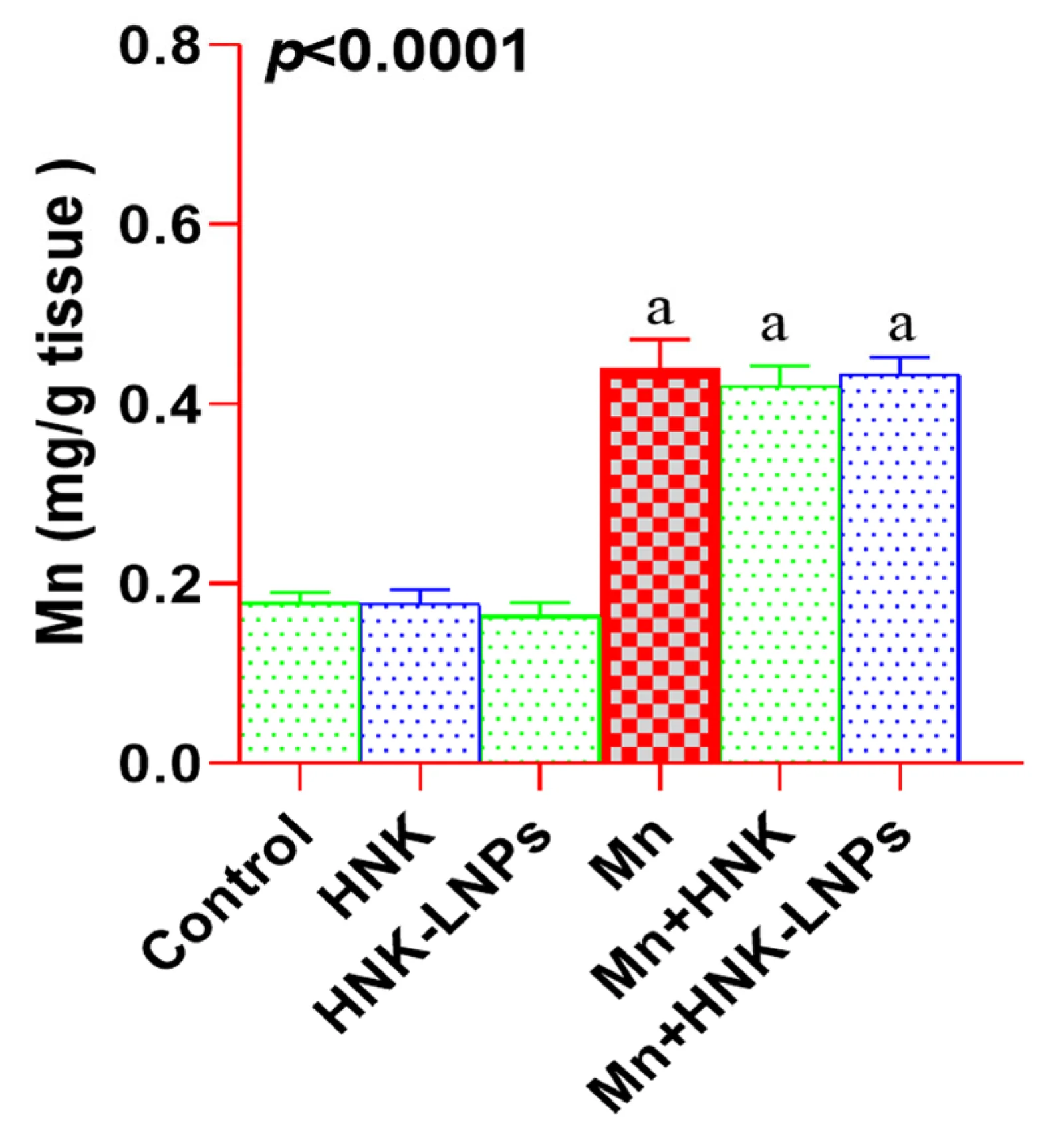
Effect of honokiol (HNK) and HNK-LNPs on Mn concentration in hippocampal tissue of manganese (Mn)-exposed rats. Results are presented as mean ± SEM. Data were obtained from 10 independent animals per group (n = 10 biological replicates/group). Each biological sample was assayed in duplicate (two technical replicates), and the mean of the duplicate measurements was used as the individual animal value for statistical analysis. a: denotes significant differences (*p* < 0.05) relative to the control group.

Mn concentration in the hippocampal tissue was significantly increased in the Mn group compared with the control group (*p* < 0.05). Co-treatment with either crude HNK or its nanoliposomal form (HNK-LNPs) did not produce a significant change in hippocampal Mn concentration compared with the Mn-treated group, as illustrated in Figure 4.

### 3.6. Effect of HNK Formulations on NRF2/HO-1 Signaling and Oxidative Stress

**Figure 5:**
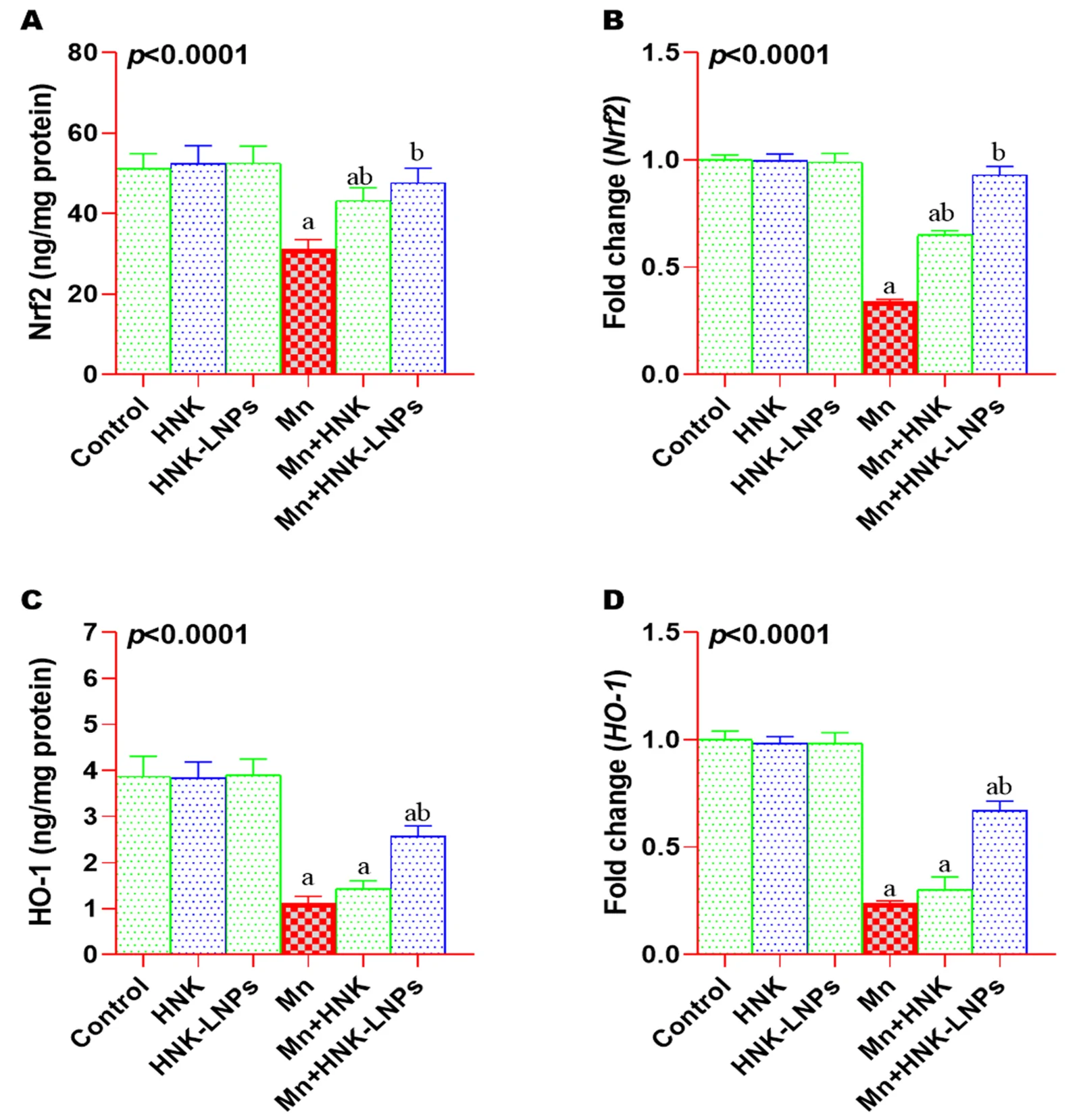
Effect of honokiol (HNK) and HNK-LNPs on the NRF2/HO-1 signaling pathway in hippocampal tissue of manganese (Mn)-exposed rats: (**A**) NRF2 protein level, (**B**) NRF2 gene expression, (**C**) HO-1 protein level, and (**D**) HO-1 gene expression. Results are presented as mean ± SEM. Data were obtained from 10 independent animals per group (n = 10 biological replicates/group). Each biological sample was assayed in duplicate (two technical replicates), and the mean of the duplicate measurements was used as the individual animal value for statistical analysis. Different superscripts (a and b) denote significant differences (*p* < 0.05) relative to the control group (a) and the Mn group (b).

Mn exposure markedly suppressed the NRF2/HO-1 antioxidant axis, as reflected by significant reductions in NRF2 protein concentration (Figure 5A) and mRNA expression (Figure 5B), as well as HO-1 protein (Figure 5C) and gene expression (Figure 5D), relative to control animals. Co-treatment with either HNK formulation or Mn significantly restored NRF2 levels at both the protein and transcriptional levels, with the most pronounced recovery observed in the Mn+HNK-LNPs group, whose NRF2 levels were statistically indistinguishable from those of the control. HO-1 protein and gene expression were similarly elevated by HNK-LNPs co-treatment, demonstrating a clear advantage over free HNK; the latter produced no statistically significant change relative to the Mn-only group at either molecular level.

**Figure 6:**
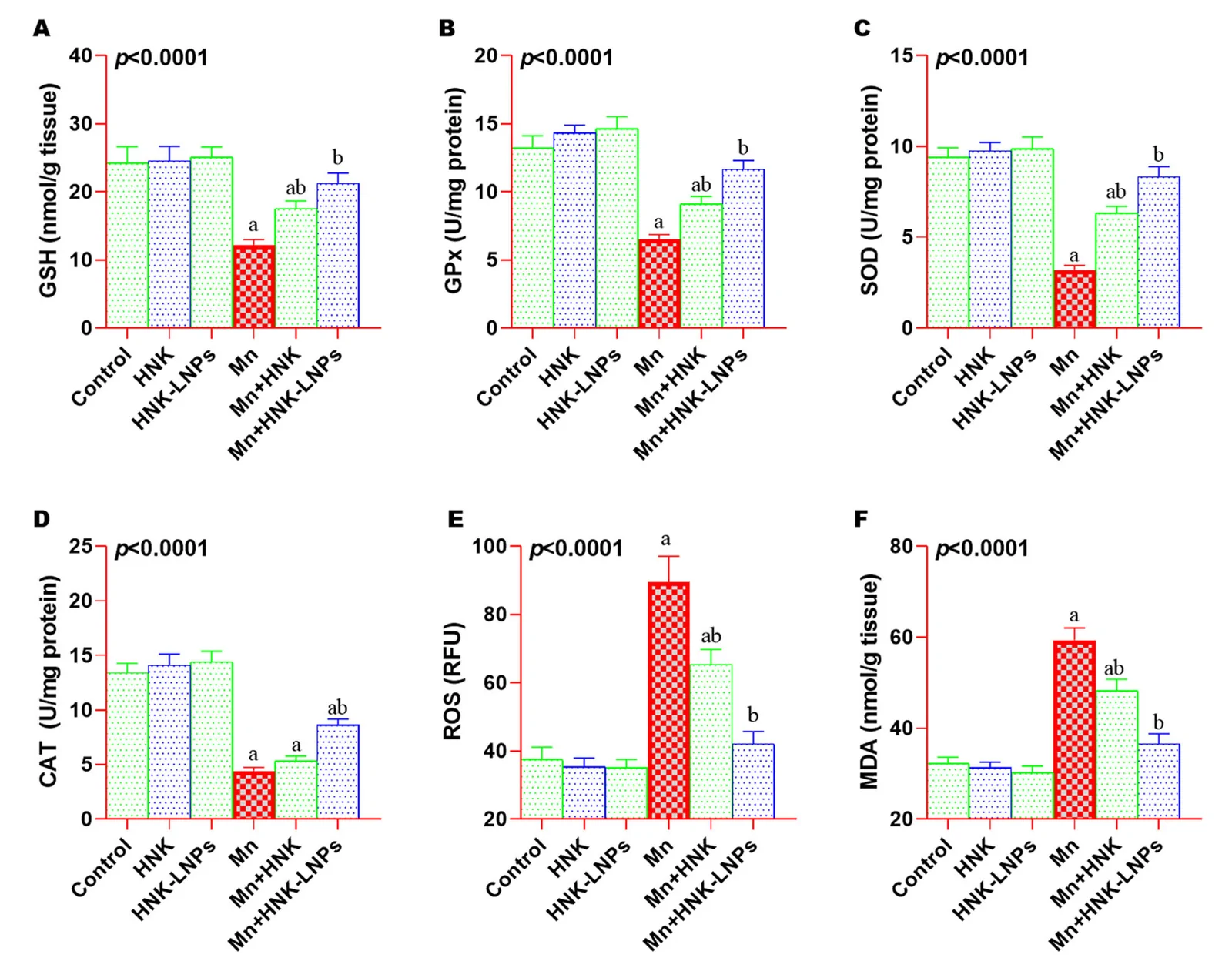
Effect of honokiol (HNK) and HNK-LNPs on antioxidant status and oxidative stress biomarkers in hippocampal tissue of Mn-exposed rats: (**A**) GSH (reduced glutathione), (**B**) GPx activity (glutathione peroxidase), (**C**) SOD activity (superoxide dismutase), (**D**) CAT activity (catalase), (**E**) ROS level (reactive oxygen species), and (**F**) MDA level (malondialdehyde). Results are presented as mean ± SEM. Data were obtained from 10 independent animals per group (n = 10 biological replicates/group). Each biological sample was assayed in duplicate (two technical replicates), and the mean of the duplicate measurements was used as the individual animal value for statistical analysis. Different superscripts (a and b) denote significant differences (*p* < 0.05) relative to the control group (a) and the Mn group (b).

Mn exposure concurrently disrupted the hippocampal antioxidant defense system, producing significant decreases in reduced glutathione (GSH; Figure 6A) and marked reductions in the activities of GPx (Figure 6B), SOD (Figure 6C), and CAT (Figure 6D) relative to controls. Co-treatment with both HNK formulations significantly improved GSH levels and GPx and SOD activities, with values in the Mn+HNK-LNPs group restored to control levels. CAT activity was significantly elevated in the Mn+HNK-LNPs group relative to the Mn-only group, whereas free HNK co-treatment produced no statistically significant change in CAT activity. Hippocampal ROS (Figure 6E) and MDA (Figure 6F) were significantly elevated by Mn exposure; HNK-LNPs co-treatment produced significant reductions in both markers, restoring them to control-comparable levels, whereas free HNK exhibited a comparatively attenuated effect.

### 3.7. Effect of HNK Formulations on Neuroinflammatory Markers

**Figure 7:**
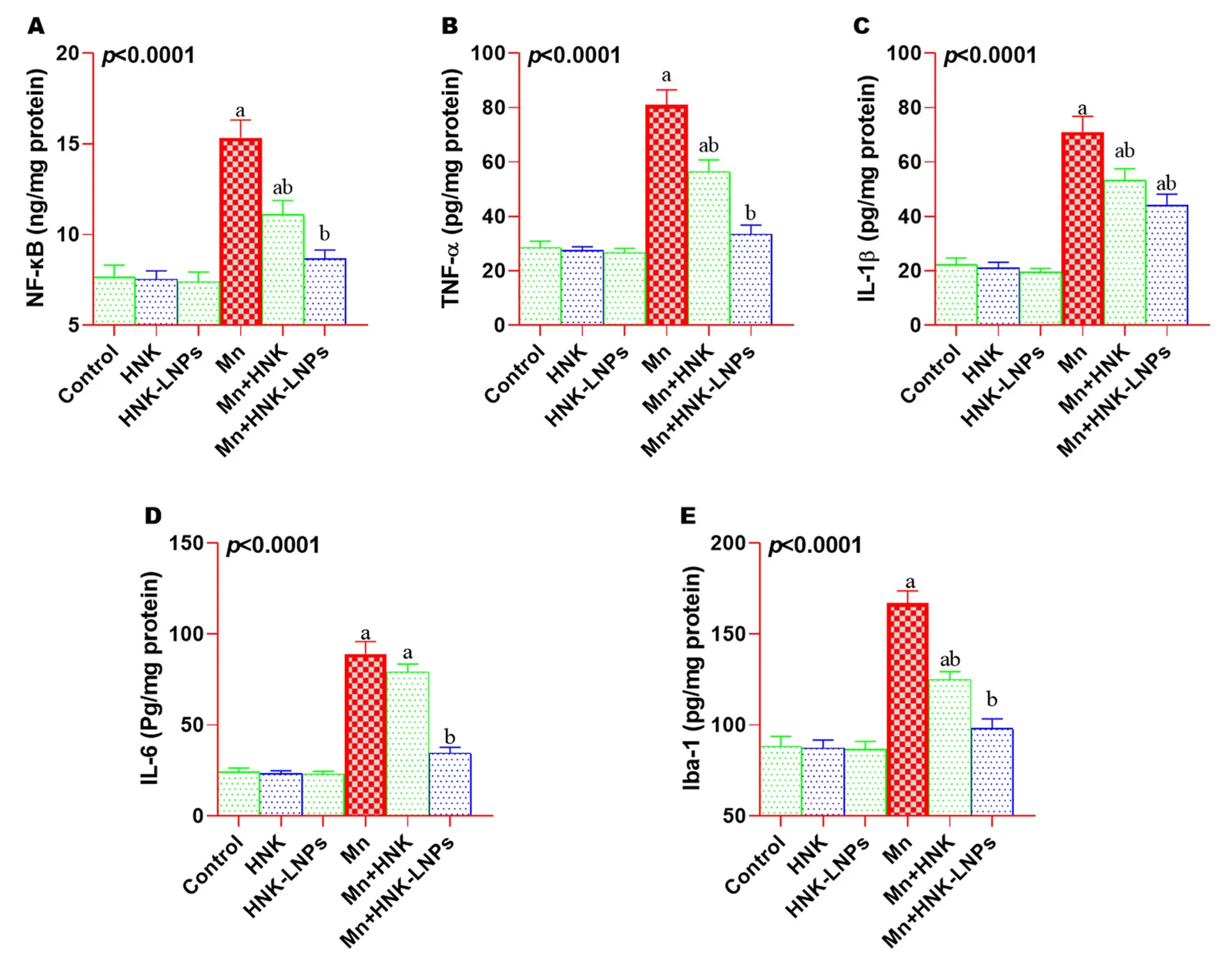
Effect of honokiol (HNK) and HNK-LNPs on neuroinflammatory markers in hippocampal tissue of Mn-exposed rats: (**A**) NF-κB, (**B**) TNF-α (tumor necrosis factor-alpha), (**C**) IL-1β (interleukin-1 beta), (**D**) IL-6 (interleukin-6), and (**E**) Iba-1 (ionized calcium-binding adapter molecule 1). Results are presented as mean ± SEM. Data were obtained from 10 independent animals per group (n = 10 biological replicates/group). Each biological sample was assayed in duplicate (two technical replicates), and the mean of the duplicate measurements was used as the individual animal value for statistical analysis. Different superscripts (a and b) denote significant differences (*p* < 0.05) relative to the control group (a) and the Mn group (b).

Mn exposure produced a robust neuroinflammatory response in the hippocampus, evidenced by significant elevations in NF-κB (Figure 7A), TNF-α (Figure 7B), IL-1β (Figure 7C), and IL-6 (Figure 7D) relative to controls. Co-treatment with either HNK formulation significantly attenuated NF-κB, TNF-α, and IL-1β levels compared with the Mn-only group, with Mn+HNK-LNPs producing the greatest reductions, reaching control-comparable levels for NF-κB and TNF-α. For IL-6, free HNK co-treatment failed to produce a statistically significant reduction relative to the Mn group, whereas HNK-LNPs co-treatment achieved a significant decrease. Altered microglia-associated Iba-1 signaling (Figure 7E) was significantly heightened following Mn exposure. Both HNK formulations significantly reduced hippocampal Iba-1 levels, with HNK-LNPs co-treatment yielding the most pronounced attenuation, reducing values to control-comparable levels.

### 3.8. Effect of HNK Formulations on Hippocampal Neurotransmitter Levels

**Figure 8:**
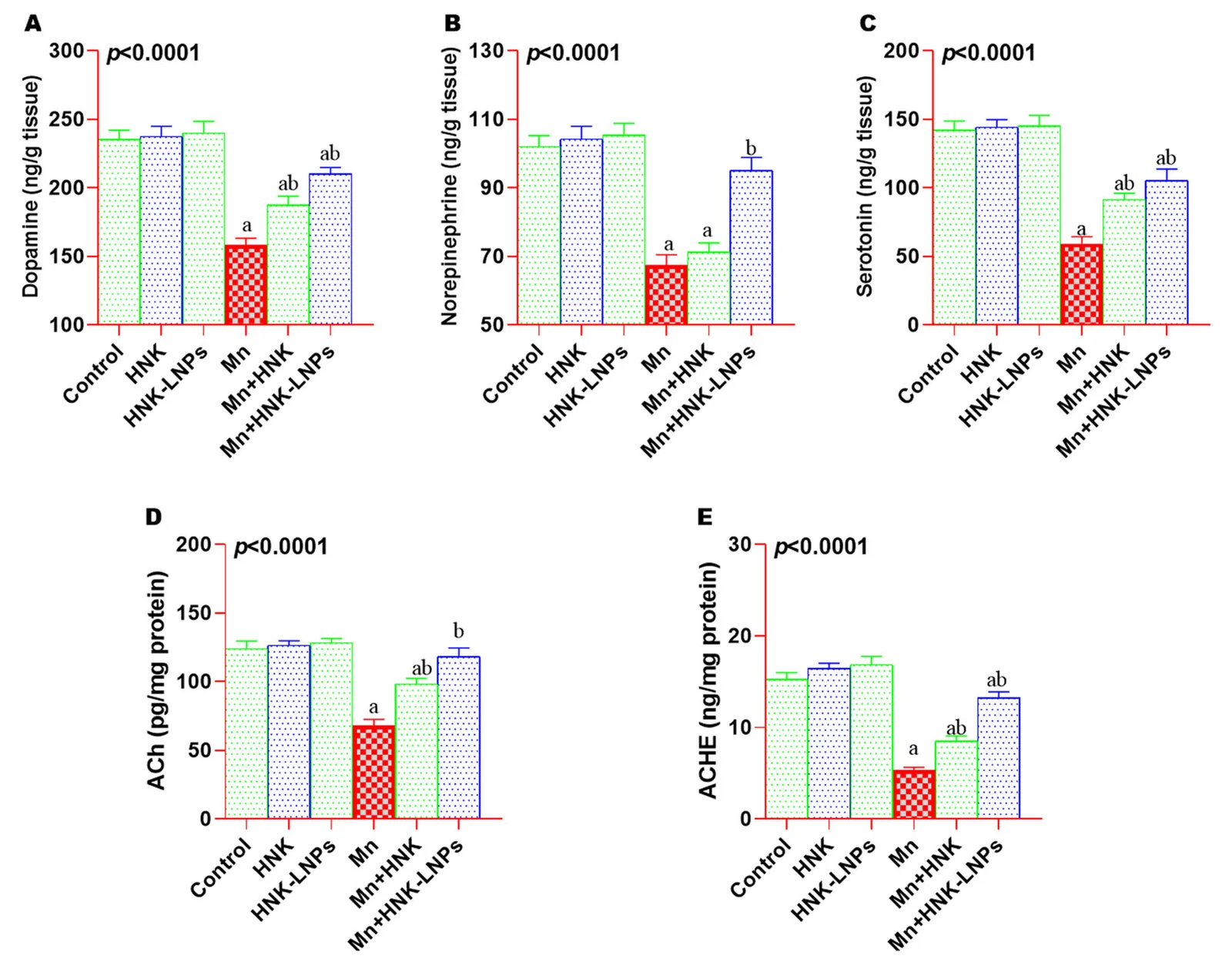
Effect of honokiol (HNK) and HNK-LNPs on hippocampal neurotransmitter profiles in Mn-exposed rats: (**A**) dopamine, (**B**) norepinephrine, (**C**) serotonin, (**D**) acetylcholine (ACh), and (**E**) acetylcholinesterase (AChE) activity. Results are presented as mean ± SEM. Data were obtained from 10 independent animals per group (n = 10 biological replicates/group). Each biological sample was assayed in duplicate (two technical replicates), and the mean of the duplicate measurements was used as the individual animal value for statistical analysis. Different superscripts (a and b) denote significant differences (*p* < 0.05) relative to the control group (a) and the Mn group (b).

Mn exposure significantly depleted hippocampal monoamine concentrations, with marked reductions in dopamine (Figure 8A), norepinephrine (Figure 8B), and serotonin (Figure 8C) relative to controls. HNK-LNPs co-treatment produced a significantly greater restoration of all three monoamines compared with free HNK, with norepinephrine levels in the Mn+HNK-LNPs group reaching levels statistically comparable to those in controls. Cholinergic neurotransmission was similarly impaired, with significant reductions in both ACh concentration (Figure 8D) and AChE activity (Figure 8E) observed after Mn exposure. Co-treatment with either formulation significantly improved both parameters, with HNK-LNPs demonstrating superior efficacy; ACh levels in the nanoformulation group were restored to values indistinguishable from those of the control.

### 3.9. Effect of HNK Formulations on Mitochondrial Respiratory Chain Function and Energy Metabolism

**Figure 9:**
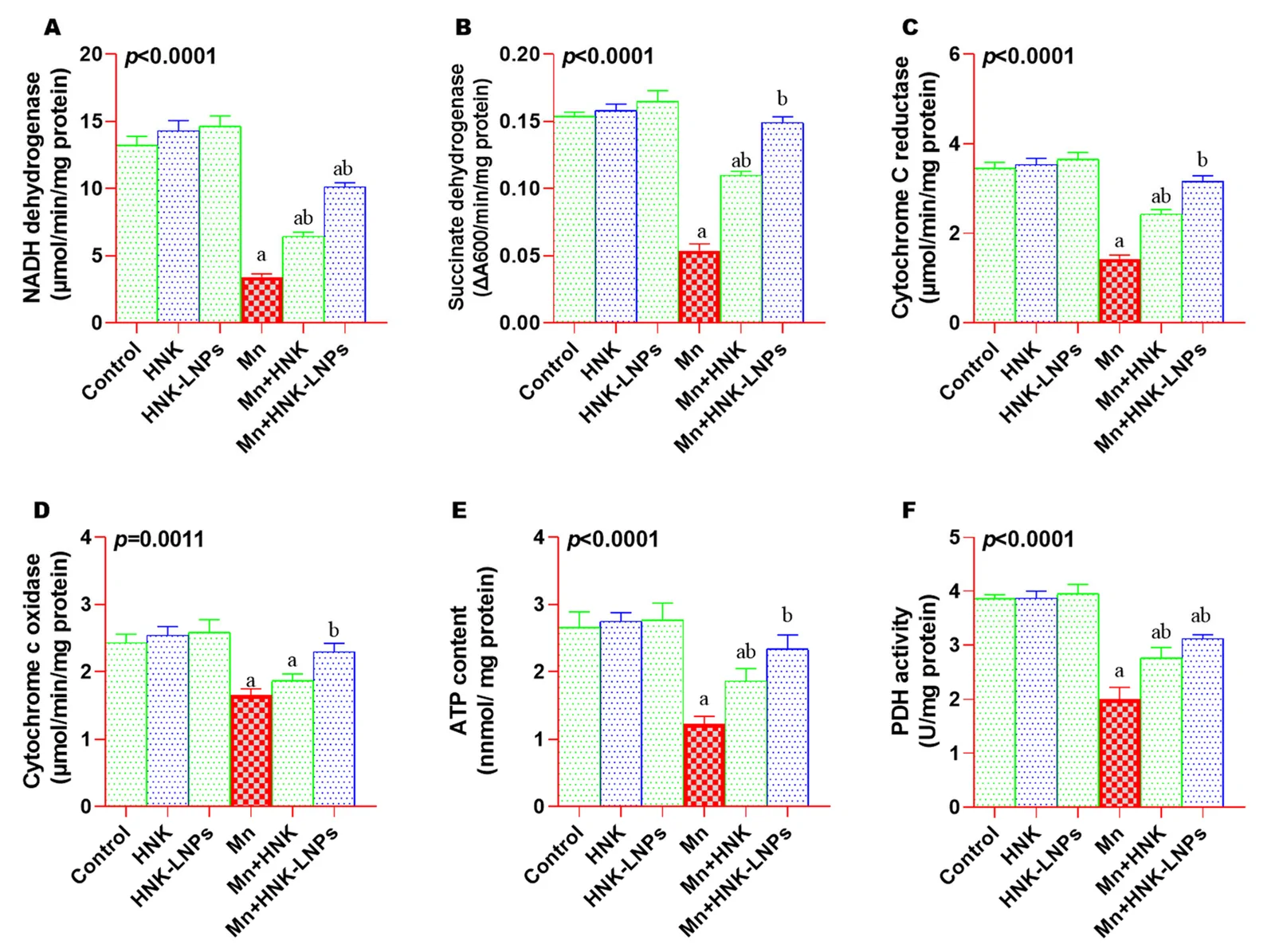
Effect of honokiol (HNK) and HNK-LNPs on mitochondrial respiratory chain complexes and cellular energy metabolism in hippocampal tissue of Mn-exposed rats: (**A**) Complex I (NADH dehydrogenase), (**B**) Complex II (succinate dehydrogenase), (**C**) Complex III (cytochrome c reductase), (**D**) Complex IV (cytochrome c oxidase), (**E**) ATP levels, and (**F**) PDH (pyruvate dehydrogenase) activity. Results are presented as mean ± SEM. Data were obtained from 10 independent animals per group (n = 10 biological replicates/group). Each biological sample was assayed in duplicate (two technical replicates), and the mean of the duplicate measurements was used as the individual animal value for statistical analysis. Different superscripts (a and b) denote significant differences (*p* < 0.05) relative to the control group (a) and the Mn group (b).

Mn exposure significantly impaired all four mitochondrial respiratory chain complexes, producing marked reductions in the activities of NADH dehydrogenase (Complex I; Figure 9A), succinate dehydrogenase (Complex II; Figure 9B), cytochrome c reductase (Complex III; Figure 9C), and cytochrome c oxidase (Complex IV; Figure 9D) relative to controls. Co-treatment with either HNK formulation significantly restored Complex I, II, and III activities, with HNK-LNPs yielding values statistically comparable to controls for all three complexes. Complex IV activity was significantly improved only in the Mn+HNK-LNPs group, while free HNK co-treatment produced no statistically significant change relative to the Mn-only group. Reflecting the downstream bioenergetic consequences of these mitochondrial insults, both ATP levels (Figure 9E) and pyruvate dehydrogenase (PDH) activity (Figure 9F) were significantly reduced by Mn exposure. Both HNK formulations significantly improved these parameters, with HNK-LNPs demonstrating superior efficacy; ATP levels in the nanoformulation group recovered to levels comparable to those of controls.

### 3.10. Effect of HNK Formulations on SIRT1/PGC-1α Signaling

**Figure 10:**
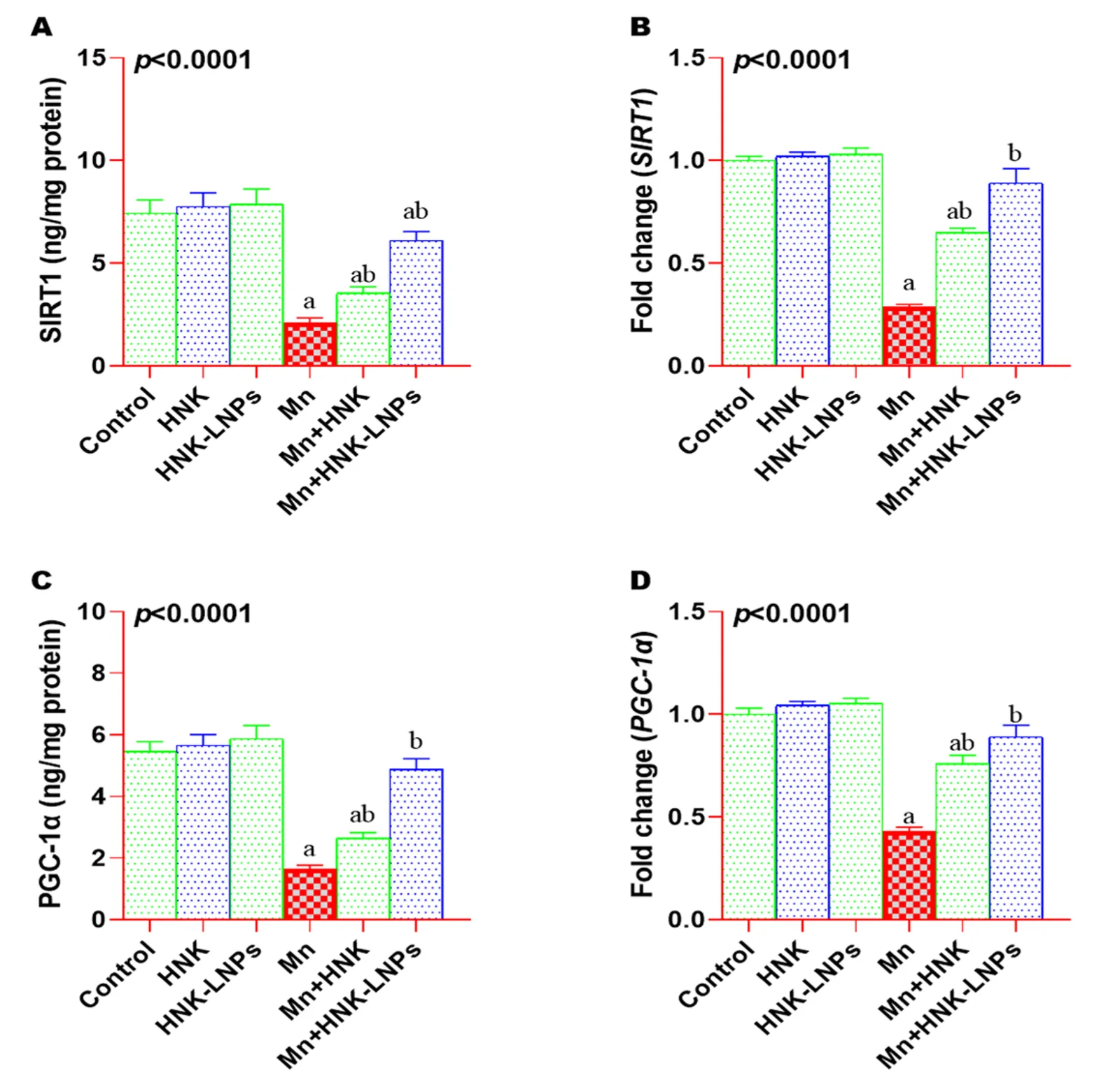
Effect of honokiol (HNK) and HNK-LNPs on the SIRT1/PGC-1α signaling pathway in hippocampal tissue of Mn-exposed rats: (**A**) SIRT1 protein level (sirtuin 1), (**B**) SIRT1 gene expression, (**C**) PGC-1α protein level (peroxisome proliferator-activated receptor gamma coactivator-1 alpha), and (**D**) PGC-1α gene expression. Results are presented as mean ± SEM. Data were obtained from 10 independent animals per group (n = 10 biological replicates/group). Each biological sample was assayed in duplicate (two technical replicates), and the mean of the duplicate measurements was used as the individual animal value for statistical analysis. Different superscripts (a and b) denote significant differences (*p* < 0.05) relative to the control group (a) and the Mn group (b).

Mn exposure significantly downregulated SIRT1/PGC-1α signaling in the hippocampus, as evidenced by marked reductions in SIRT1 protein (Figure 10A) and mRNA expression (Figure 10B), as well as PGC-1α protein (Figure 10C) and gene expression (Figure 10D), relative to controls. Co-treatment with either HNK formulation significantly upregulated this pathway, with HNK-LNPs exerting the most pronounced effect. SIRT1 mRNA expression and both PGC-1α protein and gene expression in the Mn+HNK-LNPs group were restored to levels statistically indistinguishable from those of the control.

### 3.11. Effect of HNK Formulations on Apoptotic Signaling

**Figure 11:**
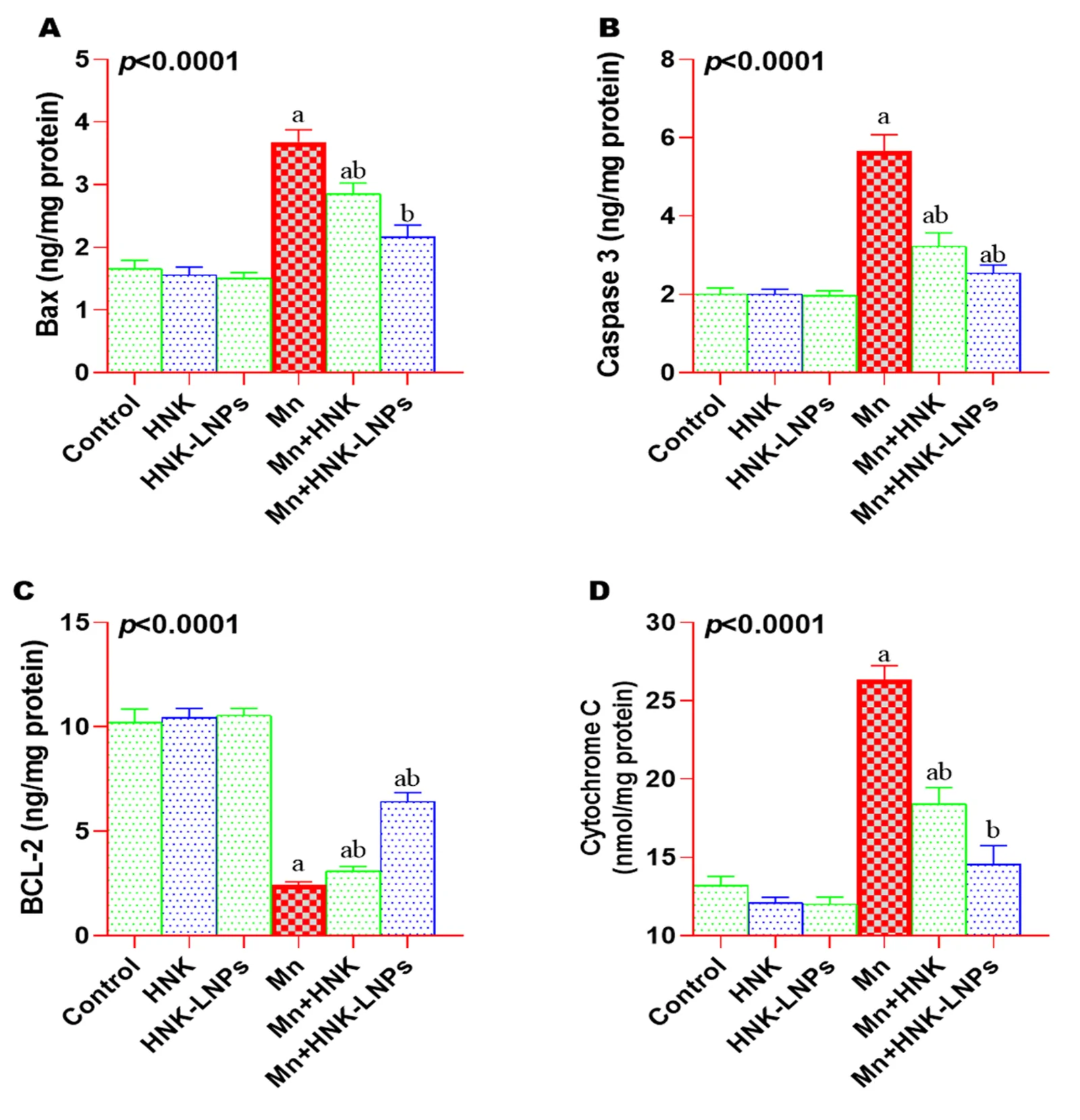
Effect of honokiol (HNK) and HNK-LNPs on apoptotic markers in the hippocampal tissue of manganese (Mn)-exposed rats, showing (**A**) Bax level (Bcl-2-associated X protein), (**B**) caspase-3 level (Caspase-3), (**C**) Bcl-2 level (B-cell lymphoma 2), and (**D**) cytochrome c level (Cytochrome c). Results are shown as mean ± SEM. Data were obtained from 10 independent animals per group (n = 10 biological replicates/group). Each biological sample was assayed in duplicate (two technical replicates), and the mean of the duplicate measurements was used as the individual animal value for statistical analysis. The presence of different superscripts (a and b) signifies significant variation (*p* < 0.05) when compared to the control group (a) and the Mn group (b).

Mn exposure significantly elevated the hippocampal levels of pro-apoptotic proteins, including Bax (Figure 11A) and caspase-3 (Figure 11B), relative to controls. Co-treatment with either HNK formulation significantly attenuated both markers, with Mn+HNK-LNPs producing the greatest reductions; Bax levels in this group were restored to control levels. Conversely, the anti-apoptotic protein Bcl-2 (Figure 11C) was significantly depleted following Mn exposure. Both HNK formulations significantly elevated Bcl-2 expression, with the greatest increase achieved in the HNK-LNPs group. Cytochrome c release (Figure 11D) was significantly elevated by Mn, reflecting mitochondrial outer membrane permeabilization; HNK-LNPs co-treatment produced a significant reduction in cytochrome c levels, restoring them to values comparable to those of the control group, whereas free HNK showed a comparatively attenuated effect.

### 3.12. Histopathological Findings

**Figure 12:**
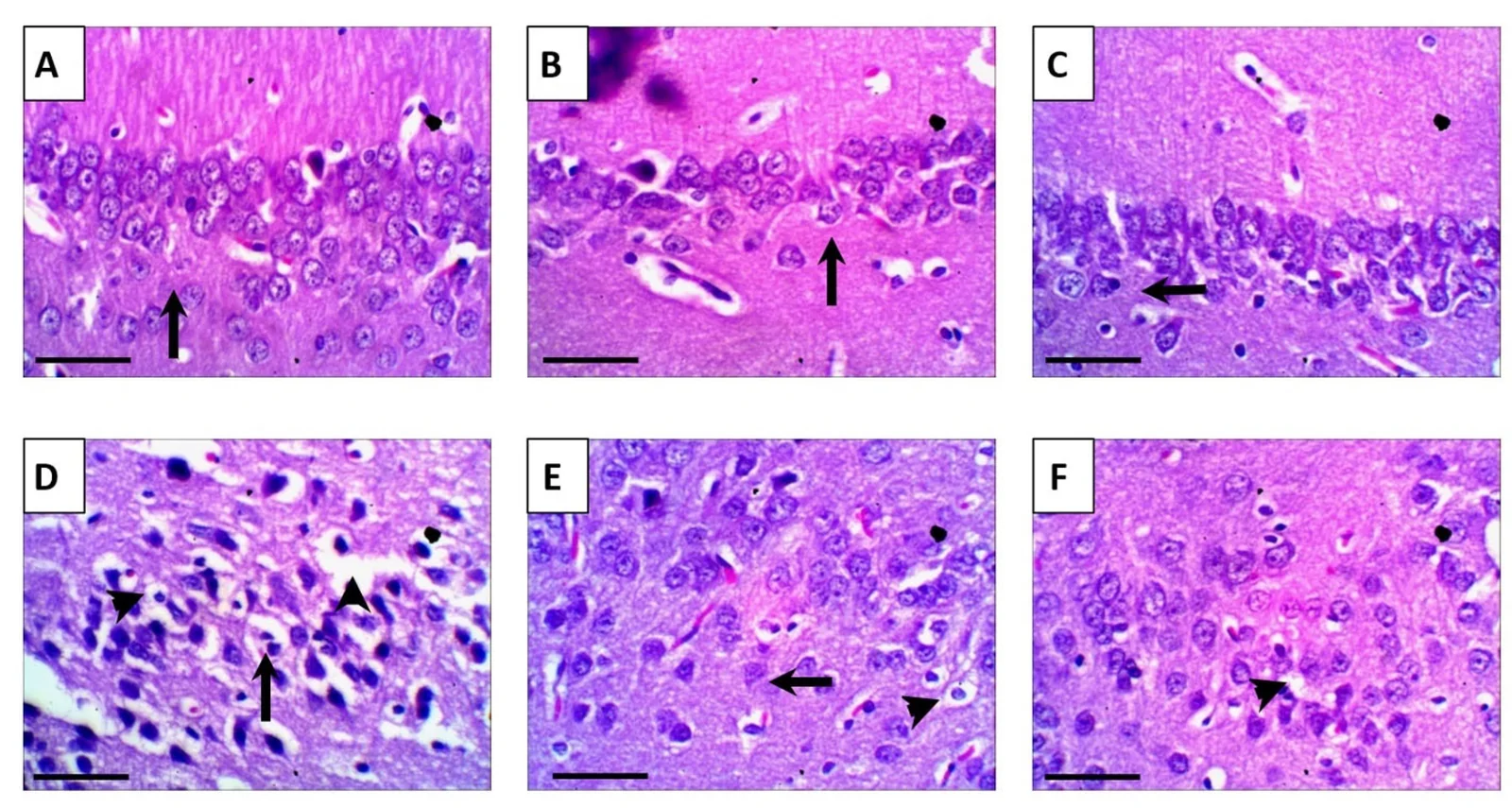
Histological sections of the hippocampus showing representative photomicrographs from: (**A**) control, (**B**) HNK, (**C**) HNK-LNPs, (**D**) Mn, (**E**) Mn+HNK, and (**F**) Mn+HNK-LNPs groups. Magnification: 400×; scale bar: 50 µm. Thin arrows indicate neuronal nuclei; arrowheads denote vacuolated neuropil. Histopathological examination was performed in 10 independent animals per group (n = 10 biological replicates/group), and representative photomicrographs are shown.

Light microscopic examination of control hippocampal sections (Figure 12A) revealed well-preserved neuronal architecture with intact, round nuclei and clearly delineated neuropil. Sections from HNK-alone (Figure 12B) and HNK-LNPs-alone (Figure 12C) groups were indistinguishable from controls, confirming the absence of treatment-related histotoxicity. By contrast, Mn-exposed hippocampi (Figure 12D) exhibited extensive neurodegeneration, characterized by shrunken, pyknotic nuclei, pronounced neuropil vacuolation, and loss of normal cellular organization. These pathological alterations were substantially ameliorated in the Mn+HNK group (Figure 12E) and most notably in the Mn+HNK-LNPs group (Figure 12F), in which neuronal morphology and tissue architecture were largely preserved, approaching the appearance of controls.

### 3.13. Ultrastructural Examination

**Figure 13:**
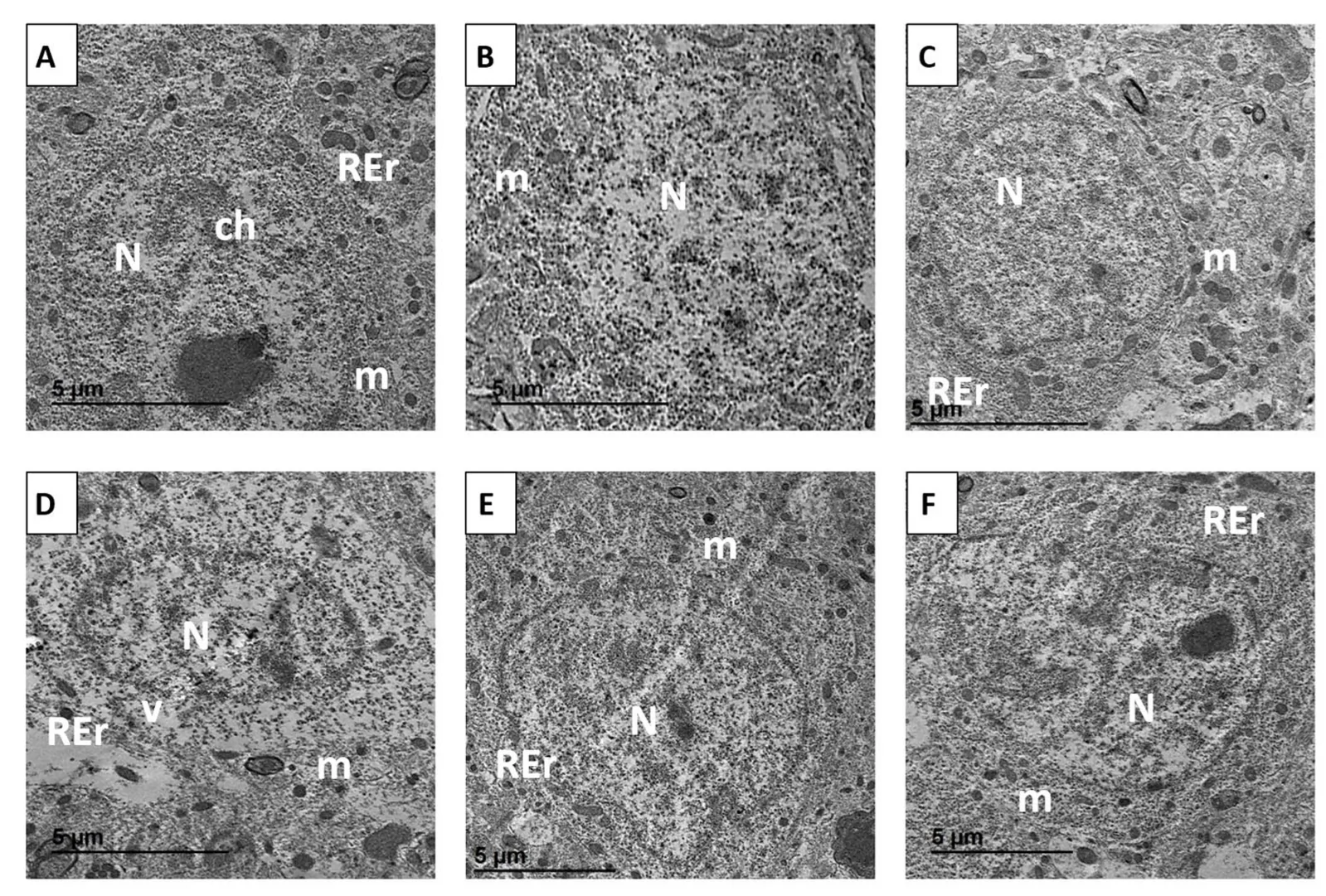
Ultrastructural features of hippocampal tissue in: (**A**) control, (**B**) HNK, (**C**) HNK-LNPs, (**D**) Mn, (**E**) Mn+HNK, and (**F**) Mn+HNK-LNPs groups. N = nucleus; m = mitochondria; REr = rough endoplasmic reticulum; ch = chromatin; v = vacuolated cytoplasm. Ultrastructural examination was performed in five independent animals per group (n = 5 biological replicates/group), and representative TEM micrographs are shown.

Hippocampal neurons from control animals (Figure 13A–C) displayed preserved ultrastructure, featuring intact nuclear membranes, uniformly dispersed euchromatin, and round nuclei. The cytoplasm contained well-organized rough endoplasmic reticulum (RER), and mitochondria exhibited clearly defined cristae with intact inner and outer membranes, collectively reflecting normal cellular homeostasis. In the Mn-treated group (Figure 13D), hippocampal neurons showed profound ultrastructural deterioration. Nuclear morphology was distorted, with irregular and dilated nuclear envelopes replacing the normal round contour. Extensive cytoplasmic vacuolation was evident, mitochondria appeared shrunken with disrupted cristae, and the RER was markedly swollen, changes collectively indicative of severe degenerative injury. In the Mn+HNK and Mn+HNK-LNPs groups (Figure 13E,F), neuronal ultrastructure was substantially recovered. Nuclei regained a predominantly normal shape with intact envelopes and evenly distributed chromatin. Cytoplasmic RER was well-maintained, mitochondrial architecture was largely restored, and vacuolation was minimal and infrequent, demonstrating meaningful cytoprotection, most evident in the nanoformulation-treated group.

### 3.14. Immunohistochemical Analysis of NF-κB in the Hippocampus

**Figure 14:**
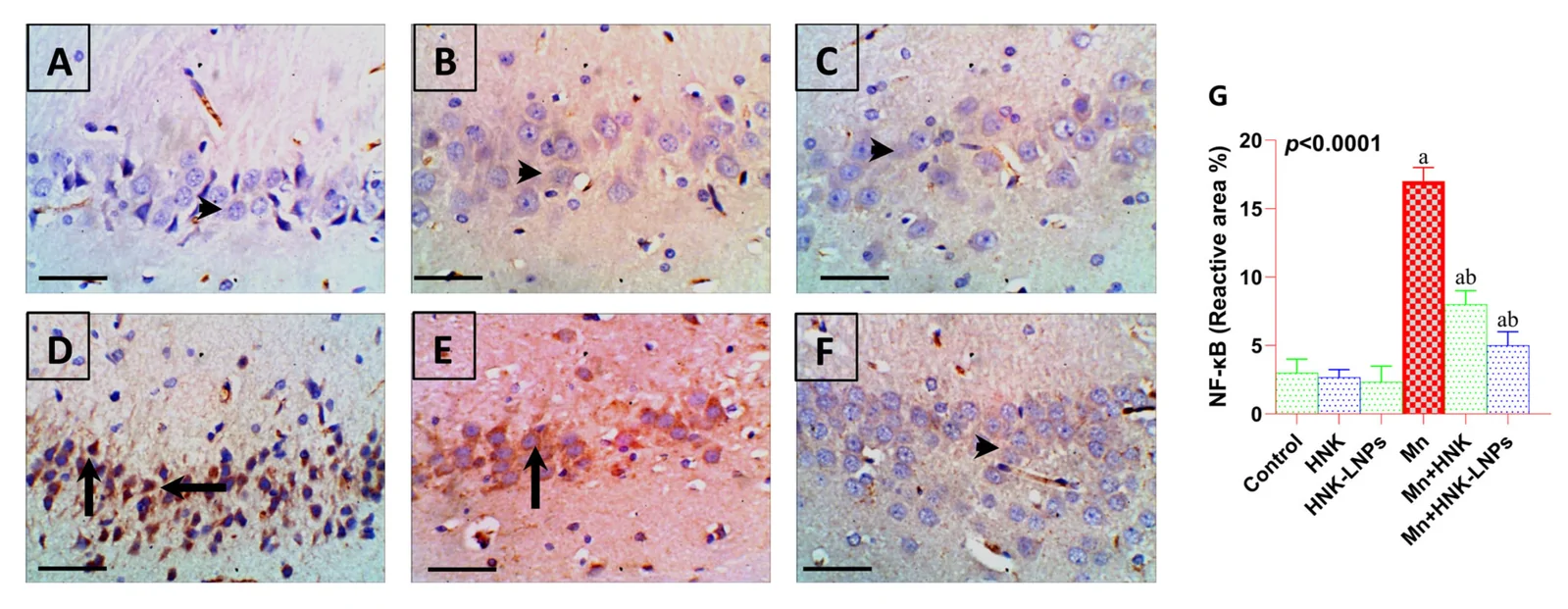
Immunohistochemical expression of NF-κB in hippocampal sections from: (**A**) control, (**B**) HNK, (**C**) HNK-LNPs, (**D**) Mn, (**E**) Mn+HNK, and (**F**) Mn+HNK-LNPs groups. Brown staining indicates NF-κB immunoreactivity. Arrows indicate NF-κB-positive cells. Arrowheads indicate NF-κB-negative cells. Scale bar = 50 µm. (**G**) Quantitative analysis of NF-κB-reactive area (%). Data are presented as mean ± SEM. Histopathological examination and quantitative analysis were performed in 10 independent animals per group (n = 10 biological replicates/group), and representative photomicrographs are shown. ‘a’ indicates a significant difference versus control and ‘b’ indicates a significant difference versus Mn (*p* < 0.05).

Immunohistochemical examination showed minimal NF-κB immunoreactivity in the control, HNK, and HNK-LNPs groups (Figure 14A–C), with no significant differences among these groups. Mn exposure markedly increased NF-κB-reactive area compared with the control (17.00% vs. 3.00%, *p* < 0.0001; Figure 14D). Co-treatment with HNK significantly reduced NF-κB immunoreactivity to 8.00% (*p* < 0.0001 vs. Mn), while HNK-LNPs produced a further reduction to 5.00% (*p* < 0.0001 vs. Mn; *p* = 0.0246 vs. Mn+HNK) (Figure 14E,F). The Mn+HNK-LNPs group did not differ significantly from the control (*p* = 0.1925), whereas the Mn+HNK group remained significantly higher (*p* = 0.0004). These quantitative findings were consistent with the representative histological staining patterns (Figure 14 G).

## 4. Discussion

Excessive accumulation of manganese in the central nervous system poses a serious neurotoxic threat, precipitating motor abnormalities, cognitive deterioration, and irreversible hippocampal neuronal injury through converging pathological mechanisms that include oxidative stress, neuroinflammation, mitochondrial impairment, apoptotic activation, and disruption of neurotransmitter homeostasis [33,34,35]. Against this background, the present study evaluated and compared the neuroprotective capacity of free HNK and its liposomal nanoformulation (HNK-LNPs) in a rat model of Mn-induced hippocampal neurotoxicity, with particular emphasis on the NRF2/HO-1 and SIRT1/PGC-1α molecular axes.

### 4.1. Oxidative Stress and Antioxidant Defense

Oxidative stress plays a central role in the pathogenesis of Mn neurotoxicity, arising from an imbalance between excessive ROS production and the erosion of the brain’s intrinsic antioxidant machinery [34,36]. Consistent with this, Mn-exposed animals in the present study exhibited significantly elevated hippocampal levels of MDA and ROS, reflecting intensified lipid peroxidation and oxidative stress on neuronal membranes and intracellular macromolecules [5,37]. Concurrently, significant depletions in GSH, GPx, CAT, and SOD were observed, indicating the breakdown of both enzymatic and non-enzymatic antioxidant tiers, which are indispensable for free radical scavenging and redox homeostasis [12]. GSH serves as the primary non-enzymatic reductant, directly quenching ROS and regenerating oxidized thiol residues [38]; GPx reduces hydroperoxides at the expense of GSH [39]; CAT converts hydrogen peroxide to oxygen and water [40]; and SOD catalyzes the conversion of superoxide anion to the less reactive hydrogen peroxide [40]. The cumulative disruption of these defenses is consistent with prior evidence demonstrating that Mn-driven redox dysregulation ultimately precipitates mitochondrial dysfunction and apoptotic cascades [11,12]. Furthermore, the observed elevation in lipid peroxidation indices aligns with emerging data linking Mn-triggered ferroptosis to sustained oxidative membrane damage and neuronal death [13].

HNK-LNPs co-treatment significantly reversed these oxidative perturbations, reducing MDA and ROS while restoring GSH levels and antioxidant enzyme activities toward control values, with greater effects than free HNK across several of these parameters. However, because blank-liposome and Mn+blank-liposome control groups were not included, a potential contribution of the liposomal carrier to these differences cannot be excluded, and the greater effects should therefore be interpreted as formulation-associated rather than attributed exclusively to encapsulated HNK. Although liposomal encapsulation may favor the physicochemical and delivery properties of hydrophobic compounds [41], the present study did not assess pharmacokinetics, brain biodistribution, hippocampal HNK concentrations, or BBB transport; therefore, the greater efficacy of HNK-LNPs cannot be attributed directly to enhanced CNS bioavailability. The improvements observed with HNK-LNPs were also associated with restoration of NRF2 and HO-1 levels, consistent with modulation of the NRF2/HO-1 antioxidant pathway [19]. These findings align with the work of Hou et al. [42], who demonstrated that honokiol shields neurons from oxidative injury through NRF2 activation and upregulation of cytoprotective enzymes, and with Zhou et al. [19], who showed that HNK alleviates oxidative burden in amyotrophic lateral sclerosis models by augmenting GSH synthesis and activating the NRF2-ARE signaling cascade.

### 4.2. Neuroinflammation and Altered Microglia-Associated Iba-1 Signaling

Neuroinflammation represents a self-perpetuating amplifier of Mn-induced neurotoxicity, in which oxidative stress triggers increased NF-κB expression, pro-inflammatory cytokine elaboration, and microglial response, collectively intensifying neuronal injury [43]. In the present study, Mn exposure elicited significant increases in NF-κB, TNF-α, IL-1β, IL-6, and Iba-1 in the hippocampus, consistent with an enhanced neuroinflammatory response. NF-κB orchestrates the transcription of inflammatory, immune, and apoptosis-related gene networks [44], while TNF-α, IL-1β, and IL-6 propagate neuroinflammatory cascades that further compound oxidative neuronal damage [45]. The marked elevation of Iba-1, a predominantly microglia-associated marker, is consistent with an increased microglial response to Mn exposure [46]. However, because Iba-1 was quantified in whole hippocampal tissue without cell counting, morphological assessment, or cellular phenotyping, these findings cannot distinguish increased microglial abundance from altered activation state. Moreover, infiltration or changes in other innate and adaptive immune-cell populations were not specifically evaluated. Accordingly, the Iba-1 findings should be interpreted as evidence of altered microglia-associated signaling rather than definitive demonstration of microglial activation or expansion.

Co-treatment with HNK significantly attenuated NF-κB activation, curtailed pro-inflammatory cytokine production, and suppressed microglial reactivity, with HNK-LNPs exerting consistently superior effects. These anti-inflammatory actions are mechanistically grounded in honokiol’s capacity to stabilize IκBα, thereby preventing p65 nuclear translocation and downstream transcription of inflammatory genes [21]. In line with Chen et al., honokiol attenuated TNF-α-induced neutrophil adhesion to cerebral endothelial cells by preserving IκBα integrity and blocking NF-κB nuclear entry, providing a relevant precedent for the neuroinflammatory context of the present study [47].

### 4.3. Mitochondrial Dysfunction and Apoptosis

Mitochondria represent a primary cellular target of Mn toxicity, as the metal preferentially accumulates in the mitochondrial matrix and disrupts the integrity of the electron transport chain, bioenergetic homeostasis, and membrane potential [5]. In the current study, Mn exposure significantly impaired the activities of all four respiratory chain complexes, reduced ATP content, and decreased PDH activity, an enzyme that links glycolysis with the tricarboxylic acid cycle [48]. Dysfunction of complexes I–IV compromises the proton gradient essential for oxidative phosphorylation and promotes electron leakage, which, in turn, increases mitochondrial ROS generation [48]. This interpretation is consistent with recent evidence showing that Mn-associated oxidative stress can impair mitochondrial calcium homeostasis and respiratory function, thereby exacerbating ROS generation, bioenergetic failure, and susceptibility to apoptosis [35]. These bioenergetic failures were accompanied by a shift toward pro-apoptotic signaling, evidenced by elevated Bax expression, cytochrome c release into the cytosol, and caspase-3 activation, alongside a reduction in the anti-apoptotic protein Bcl-2, findings collectively consistent with activation of the intrinsic mitochondrial apoptotic pathway [49].

HNK-LNPs co-treatment effectively restored mitochondrial respiratory complex activities, normalized ATP content and PDH function, and shifted the apoptotic balance toward cell survival by augmenting Bcl-2 and suppressing Bax/caspase-3/cytochrome c. HNK-LNPs showed greater efficacy than free HNK in restoring mitochondrial and apoptosis-related parameters; however, the present study did not directly assess neuronal uptake, intracellular HNK concentrations, or mitochondrial drug delivery, and therefore the basis for this difference remains to be established [19,42]. Moreover, because blank-liposome and Mn+blank-liposome control groups were not included, a potential contribution of the liposomal carrier to the observed effects cannot be excluded. Accordingly, the greater effects observed with HNK-LNPs should be interpreted as formulation-associated rather than attributed exclusively to encapsulated HNK. These observations are corroborated by Li et al. [20], who reported that honokiol improves mitochondrial function, attenuates mitochondrial ROS production, preserves mitochondrial membrane potential, and reduces neuronal apoptosis through SIRT3-mediated mitochondrial autophagy, highlighting a convergent mitochondria-protective mechanism.

Histopathological assessment revealed pronounced Mn-induced hippocampal neurodegeneration, characterized by cytoplasmic vacuolization and widespread cell degeneration. Ultrastructural examination by TEM corroborated these findings, disclosing disrupted mitochondrial architecture, swollen RER, nuclear condensation, and reactive gliosis, structural sequelae consistent with the observed biochemical profile of heightened oxidative stress, attenuated antioxidant defenses, and amplified inflammatory signaling. Treatment with HNK, most effectively in liposomal form, substantially preserved hippocampal cytoarchitecture and restored mitochondrial and ER ultrastructural integrity, thereby reducing neurodegeneration and gliosis.

### 4.4. SIRT1/PGC-1α Signaling and Neurotransmitter Homeostasis

The SIRT1/PGC-1α pathway is a pivotal regulator of mitochondrial biogenesis, oxidative metabolism, and antioxidant gene expression [14,20]. Mn exposure markedly suppressed both SIRT1 and PGC-1α at the protein and transcriptional levels, impairing the cell’s capacity to renew its mitochondrial pool and sustain metabolic resilience. HNK treatment, particularly via nanoliposomal delivery, significantly reinstated SIRT1/PGC-1α signaling, suggesting that honokiol provides not only acute neuronal protection through antioxidant and anti-apoptotic mechanisms, but also longer-term neuroprotective benefits by promoting mitochondrial biogenesis and metabolic recovery [50].

The restoration of mitochondrial function by HNK-LNPs was paralleled by a significant recovery of hippocampal neurotransmitter balance, which is critically dependent on adequate bioenergetic supply. Mn exposure produced significant reductions in dopamine, norepinephrine, serotonin, ACh, and AChE activity, perturbations that underlie the excitotoxicity, cholinergic dysfunction, and Parkinsonian-like cognitive and motor impairments characteristic of manganism [15]. Dopamine, norepinephrine, and serotonin govern motor coordination and affective regulation [51], while acetylcholine is indispensable for hippocampus-dependent memory and cognitive function [52]. HNK-LNPs effectively reinstated monoamine and cholinergic neurotransmitter profiles, restored the inhibitory/excitatory balance, and normalized AChE activity, effects that likely reflect the compound’s upstream mitochondrial and antioxidant actions rather than direct modulation of neurotransmitter synthesis.

It is important to acknowledge that the biomarkers evaluated in the present study are biologically dynamic and may exhibit substantial variability according to Mn dose, exposure duration, brain region, cell type, timing of tissue collection, and the relative balance between injury-related and compensatory responses. For example, the reductions in HO-1 and CAT observed after Mn exposure are consistent with failure or exhaustion of inducible and enzymatic antioxidant defenses under sustained oxidative challenge [53]. However, HO-1 and CAT may increase transiently in other experimental settings as compensatory responses to early ROS generation; thus, the direction and magnitude of their changes should be interpreted in the context of the exposure paradigm and the overall redox profile [54]. In the present model, their reduction occurred concurrently with increased ROS and MDA and depletion of GSH, GPx, and SOD, supporting an overall state of impaired antioxidant capacity rather than an effective adaptive response [55].

Similarly, the increase in IL-1β should be interpreted as part of a broader, variable neuroinflammatory response rather than as an isolated event. Cytokine concentrations can fluctuate markedly over time and may depend on the activation state and relative abundance of microglia, astrocytes, infiltrating immune cells, and injured neurons [56]. The parallel increases in NF-κB, TNF-α, IL-6, and Iba-1 in the Mn-exposed group support the interpretation that elevated IL-1β reflected coordinated activation of an inflammatory milieu in the hippocampus [57]. Nevertheless, because the present measurements were performed in whole hippocampal homogenates at a single terminal time point, they cannot define the temporal sequence of cytokine induction or identify the cellular source of IL-1β.

The decrease in serotonin, together with reductions in dopamine, norepinephrine, acetylcholine, and AChE activity, is also compatible with Mn-associated impairment of neurotransmitter synthesis, vesicular handling, release, metabolism, or neuronal integrity [58,59,60,61]. However, monoamine levels are inherently susceptible to biological and analytical variability, including circadian timing, stress during handling, regional sampling, and differences in neuronal terminal preservation [62,63,64]. Therefore, the observed serotonergic deficit should be interpreted as evidence of disrupted hippocampal neurotransmitter homeostasis within the present experimental conditions, rather than proof of a direct and selective effect of Mn on serotonin synthesis.

Regarding bioenergetic biomarkers, the decreases in Complex I activity and PDH activity are mechanistically consistent with Mn-associated mitochondrial dysfunction, because Complex I is a major site of electron leakage and ROS production, whereas PDH links glycolytic pyruvate metabolism to the tricarboxylic acid cycle. Nonetheless, mitochondrial enzyme activities can differ according to mitochondrial content, tissue handling, assay conditions, substrate availability, and the stage of mitochondrial injury. The simultaneous reductions in Complexes I–IV, ATP, SIRT1/PGC-1α signaling, and PDH activity, together with ultrastructural evidence of mitochondrial damage, provide convergent support for a generalized impairment of hippocampal bioenergetics rather than an isolated alteration in a single enzyme [65,66,67].

Finally, the elevation of Bax and reduction in Bcl-2 are consistent with a shift toward mitochondrial apoptotic susceptibility, but the Bax/Bcl-2 balance is also influenced by the timing and severity of cellular stress and by heterogeneity among neuronal and glial populations [68,69]. The concurrent increase in cytochrome c and caspase-3 supports activation of the intrinsic apoptotic pathway in the Mn-exposed hippocampus [12,70]. Because these markers were measured at one time point in whole tissue, the present data indicate an apoptosis-associated molecular profile but do not quantify the proportion of individual cells undergoing apoptosis or distinguish apoptotic signaling in neurons from that in glia [71]. Future longitudinal and cell-specific studies, ideally complemented by assays such as TUNEL staining, cleaved caspase-3 immunolocalization, and mitochondrial functional analyses, would help define the temporal and cellular sources of variability in these biomarkers.

It is worth noting that although HNK and HNK-LNPs showed neuroprotective effects under the present experimental conditions, honokiol should not be regarded as universally non-toxic [72]. Its safety profile is likely to depend on dose, route of administration, formulation, exposure duration, and biological context [73]. High concentrations of honokiol have been associated with cytotoxic or developmental toxicity in selected experimental systems, and formulation-dependent changes in tissue exposure may alter both efficacy and safety [74,75,76]. Therefore, the present findings demonstrate a protective effect at the tested dose in Mn-exposed rats, but they do not establish the overall safety profile of free HNK or HNK-LNPs.

### 4.5. Limitations

The present study has several limitations that should be considered. First, behavioral outcomes, including tests of hippocampus-dependent learning and memory, locomotor activity, and motor coordination, were not included. This was because the study was prospectively designed to investigate the biochemical, molecular, mitochondrial, inflammatory, apoptotic, histopathological, and ultrastructural mechanisms underlying Mn-induced hippocampal neurotoxicity and the effects of free HNK and HNK-LNPs, rather than to evaluate functional or behavioral efficacy. Consequently, the present findings provide mechanistic and structural evidence of neuroprotection but do not establish whether these changes translate into improved cognitive, affective, or motor performance. This functional dimension has been addressed in complementary Mn-neurotoxicity studies using behavioral paradigms such as open-field, Y-maze, Morris water maze, rotarod, swimming, and catalepsy tests, which have demonstrated Mn-associated impairments in locomotion, coordination, learning, memory, and exploratory behavior. Likewise, previous preclinical studies of honokiol in other neurodegenerative and neuroinjury models have reported improvements in learning-, memory-, and exploratory-behavior outcomes. Future studies should therefore integrate validated behavioral testing with the present mechanistic endpoints to establish the functional relevance of HNK-LNP-mediated neuroprotection in Mn exposure. Although such assessments would have strengthened the functional interpretation of the findings, the specialized facilities and validated equipment required for standardized neurobehavioral testing were not available at our research facility during the study period. Second, analyses were confined to the hippocampus, whereas Mn preferentially accumulates across multiple brain regions, including the striatum and globus pallidus, which were not examined. The hippocampus was selected a priori as the principal brain region of interest in accordance with the predefined scope of the study, which focused on the biochemical, molecular, and structural features of Mn-induced hippocampal neurotoxicity. Accordingly, comprehensive assessment of additional brain regions was beyond the current experimental scope. Moreover, Mn was quantified in whole hippocampal tissue; therefore, its distribution among specific hippocampal cell types and potential effects of HNK or HNK-LNPs on cellular Mn uptake were not determined. Similarly, SOD, CAT, and GPx were assessed at the tissue level without identifying the specific neuronal or glial populations exhibiting altered antioxidant status. Future cell-specific and spatial analyses could provide further mechanistic insight into Mn neurotoxicity and the protective effects of HNK formulations. In addition, the study focused on hippocampal neurotoxicity, and potential effects of HNK or HNK-LNPs on peripheral tissues were not investigated. BBB integrity and permeability were also not directly assessed; therefore, the present findings cannot determine whether HNK or HNK-LNPs influenced Mn-induced BBB alterations or whether modulation of BBB function contributed to their observed neuroprotective effects. Third, the exclusive use of male rats limits generalization, as sex-dependent differences in Mn metabolism and neuroinflammatory responses are well documented. Fourth, no pharmacokinetic or brain biodistribution data were generated to directly substantiate the proposed bioavailability advantage of HNK-LNPs over free HNK. Furthermore, blank-liposome and Mn+blank-liposome control groups were not included; therefore, potential independent effects of the liposomal carrier on the measured biochemical, molecular, and histopathological outcomes cannot be excluded. Consequently, the greater effects observed with HNK-LNPs should be interpreted as formulation-associated rather than attributed exclusively to encapsulated HNK. Fifth, the four-week exposure protocol may not fully recapitulate the chronic, low-level Mn accumulation characteristic of occupational or environmental exposure scenarios. Future studies incorporating behavioral endpoints, broader neuroanatomical coverage, both sexes, cell-specific Mn and antioxidant assessments, BBB evaluation, peripheral-tissue assessment, and pharmacokinetic profiling would consolidate and extend these findings.

## 5. Conclusions

The present findings demonstrate that repeated Mn exposure induces marked hippocampal neurotoxicity through an interconnected triad of oxidative stress, neuroinflammation, and mitochondrial dysfunction, accompanied by suppression of the NRF2/HO-1 and SIRT1/PGC-1α cytoprotective axes, disruption of neurotransmitter homeostasis, and alterations consistent with intrinsic apoptotic signaling. These biochemical and molecular changes were associated with clear histopathological and ultrastructural evidence of progressive hippocampal neurodegeneration. Co-treatment with honokiol, particularly in its liposomal nanoparticle form, substantially mitigated these injury mechanisms, restoring antioxidant capacity, limiting neuroinflammatory activation, preserving mitochondrial bioenergetics, enhancing SIRT1/PGC-1α-linked mitochondrial biogenesis, normalizing neurotransmitter profiles, and promoting neuronal survival. HNK-LNPs consistently produced greater effects than free honokiol across the evaluated parameters under the present experimental conditions; however, because blank-liposome controls were not included, a contribution of the liposomal carrier to these differences cannot be excluded, and the greater effects should therefore be interpreted as formulation-associated rather than attributed exclusively to encapsulated HNK. Furthermore, these differences cannot be attributed to enhanced CNS bioavailability without direct pharmacokinetic and brain biodistribution evidence. These results support further investigation of HNK-LNPs as a potential strategy for mitigating Mn-induced hippocampal neurotoxicity and suggest involvement of NRF2/HO-1 and SIRT1/PGC-1α signaling in the observed protective effects. Nevertheless, extrapolation from rodents to humans should be made with caution, as pharmacokinetics, blood–brain barrier properties, and honokiol metabolism may differ between species; future studies incorporating blank-liposome controls, pharmacokinetic and brain biodistribution analyses, followed by appropriate translational investigations, are required to establish the specific contribution and clinical potential of HNK-LNPs.

## Data Availability

The original contributions presented in this study are included in the article material. Further inquiries can be directed to the corresponding author.

## Supplementary Materials

The following supporting information can be downloaded at: https://www.mdpi.com/article/10.3390/brainsci16090900/s1, Table S1: Primer sequences of the study genes.

## Abbreviations

The following abbreviations are used in this manuscript:

AAS	Atomic absorption spectrophotometry
ACh	Acetylcholine
AChE	Acetylcholinesterase
ANOVA	Analysis of variance
ATP	Adenosine triphosphate
Bax	Bcl-2-associated X protein
BCA	Bicinchoninic acid
BBB	Blood–brain barrier
Bcl-2	B-cell lymphoma 2
CAT	Catalase
CNS	Central nervous system
DCIP	2,6-Dichlorophenolindophenol
DLS	Dynamic light scattering
ELISA	Enzyme-linked immunosorbent assay
FTIR	Fourier-transform infrared spectroscopy
GAPDH	Glyceraldehyde-3-phosphate dehydrogenase
GPx	Glutathione peroxidase
GSH	Reduced glutathione
HE	Hematoxylin and eosin
HNK	Honokiol
HNK-LNPs	Honokiol-loaded liposomal nanoparticles
HPLC-ECD	High-performance liquid chromatography with electrochemical detection
HO-1	Heme oxygenase-1
Iba-1	Ionized calcium-binding adapter molecule 1
IL-1β	Interleukin-1 beta
IL-6	Interleukin-6
L-PC	L-α-phosphatidylcholine
MDA	Malondialdehyde
MIQE	Minimum information for publication of quantitative real-time PCR experiments
Mn	Manganese
NF-κB	Nuclear factor kappa-light-chain-enhancer of activated B cells
NRF2	Nuclear factor erythroid 2-related factor 2
PBS	Phosphate-buffered saline
PDI	Polydispersity index
PGC-1α	Peroxisome proliferator-activated receptor gamma coactivator-1 alpha
PDH	Pyruvate dehydrogenase
RER	Rough endoplasmic reticulum
ROS	Reactive oxygen species
RT-qPCR	Reverse transcription quantitative polymerase chain reaction
SEM	Standard error of the mean
SIRT1	Sirtuin 1
SOD	Superoxide dismutase
TEM	Transmission electron microscopy
