# Supplementary material for: Liposomal Honokiol Nanoparticles Attenuate Manganese-Induced Hippocampal Neurotoxicity via NRF2/HO-1 and SIRT1/PGC-1α Pathways: Association with Oxidative Stress, Neuroinflammation, Mitochondrial Dysfunction, and Apoptosis
Source: Brain Sci. 2026 Aug 22;16(9):900. doi: 10.3390/brainsci16090900 (PMC13604269; doi:10.3390/brainsci16090900)
Supplement: Supplementary file 1 [file brainsci-16-00900-s001.zip › brainsci-4471738-supplementary.pdf]

# Liposomal Honokiol Nanoparticles Attenuate Manganese-Induced Hippocampal Neurotoxicity via NRF2/HO-1 and SIRT1/PGC-1$\alpha$ Pathways: Association With Oxidative Stress, Neuroinflammation, Mitochondrial Dysfunction, and Apoptosis

**Table S1.** Primer sequences of the study genes

| Gene | (5'→3') |
|---|---|
| *Nrf2* | F: TTTGTAGATGACCATGAGTCG |
| | R: TCCTGCCAAACTTGCTCCAT |
| *HO-1* | F: ATGTCCCAGGATTTGTCCGA |
| | R: ATGGTACAAGGAGGCCATCA |
| *SIRT1* | F: CCAGATTTCAAGGCTGTTGGTTCC |
| | R: CCACAGGAACTAGAGGATAAGGCGT |
| *PGC-1$\alpha$* | F: TTCAGTGTCACCACCGAAATCCTTAT |
| | R: AGAGGATCTACTGCCTGGGGACC |
| *GAPDH* | F: TGGAGTCTACTGGCGTCTT |
| | R: TGTCATATTTCTCGTGGTTCA |

F: Forward, R: Reverse. "Nrf2: nuclear factor erythroid 2–related factor 2; HO-1: heme oxygenase-1; SIRT1: Sirtuin 1; PGC-1$\alpha$: Peroxisome proliferator-activated receptor gamma coactivator-1alpha; GAPDH: Glyceraldehyde-3-phosphate dehydrogenase, a housekeeping gene used as an internal control for normalization." Source: Primer-BLAST (https://www.ncbi.nlm.nih.gov/tools/primer-blast/).
